# Identifying and mapping key relationships and communication pathways influencing farmers’ antibiotic use in production animals: a scoping review

**DOI:** 10.1186/s42522-026-00215-6

**Published:** 2026-05-26

**Authors:** Carly Ching, Fiona Emdin, Sahran Shafaque, Muhammad H. Zaman, Veronika J. Wirtz

**Affiliations:** 1 Department of Biomedical Engineering, Boston University, 44 Cummington Mall, Boston, MA 02215 USA; 2 Global Strategy Lab, Toronto, ON Canada; 3 Center on Forced Displacement, Boston University, Boston, MA USA; 4 Department of Global Health, Boston University School of Public Health, Boston, MA USA

**Keywords:** One health, Antimicrobial resistance, Antibiotic stewardship

## Abstract

**Background:**

Antibiotic use in food-producing animals can drive antimicrobial resistance (AMR). Communication is a central strategy to promote behavioral change. As such, there is a need to determine from whom and where farmers receive information about antibiotic use. We aim to identify communication pathways that influence farmers’ antibiotic use in food-producing animals and understand the barriers and facilitators for using these information sources.

**Methods:**

PRISMA Extension for Scoping Reviews guidelines were followed. PubMed, CAB Abstracts, ProQuest Social Science Premium Collection, and PsycINFO were searched for primary peer-reviewed studies published from 2000 to April 10^th^, 2025, in any language. We sought studies from any country, that state from whom or where farmers or farm workers get information about antibiotic use. Title and abstract, full text screening and data charting were performed by two reviewers. One researcher inductively coded points of influence, barriers and facilitators, which were thematically combined through discussion. Quantitative and qualitative analyses were then performed.

**Results:**

Our search retrieved 5925 records. After abstract review, 140 records met the inclusion criteria. After review of full texts, 75 papers were included for data charting. Overall, we identify a complex network of 25 stakeholders, all of whom can exert influence. The most mentioned actors across eligible studies were veterinarians (86.7%), followed by peers (49.3%). The major barriers to advice were access/availability (48.6%) and cost (45.9%), and the major facilitators were trust (41.7%), followed by access/availability (33.3%). Differences in mechanics and impact on antibiotic use, along with recommendations from the literature, were also summarized

**Conclusions:**

Our results suggest that knowledge and awareness about appropriate antibiotic use and AMR must permeate multiple layers of actors with differing interests. We suggest potential strategies to address barriers, including ways to reduce costs, increase access and build trust.

**Supplementary Information:**

The online version contains supplementary material available at 10.1186/s42522-026-00215-6.

## Introduction

The use of antibiotics and antimicrobials in food-producing animals may be a critical driver of antimicrobial resistance (AMR) [1]. One strategy to promote behavioral change is effective communication. In addition to effective messaging, there is a need to determine effective strategies for delivering messages surrounding antibiotic use to farmers, including identifying who can effectively provide information and guidance [2].

Farmers and livestock producers often rely on a wide range of advice when making decisions about antimicrobial use, yet the pathways through which information flows, and the actors who influence these decisions, are poorly understood [3]. Understanding these pathways is essential, as the nature and source of advice can directly shape farmers’ perceptions, trust, and ultimately their behavior. Advice may come from veterinarians, extension officers, pharmaceutical representatives, retail drug sellers, or fellow farmers, and the type and quality of information provided can vary substantially between these sources [4, 5].

Through a scoping review, we sought to identify and map key stakeholder relationships or communication pathways that influence how farmers use antibiotics in food-producing animals. We also sought to understand the barriers and facilitators for utilizing these sources of information and advice. This information will be critical to identifying points where indiscriminate antibiotic use is facilitated and developing policy interventions which target specific influential groups.

## Methods

This scoping review followed PRISMA-ScR guidelines (Supplemental File 1) [6]. The full study protocol has been registered at Open Science Framework (OSF) [7]. We have summarized the methods below and noted any deviations.

### Study design

We conducted a scoping review to identify key stakeholders and communication pathways that influence how farmers, producers, and farm workers use antibiotics in food-producing animals. The primary scoping review research questions was:

1. From whom and where do farmers, producers, or farm workers obtain information or advice about antibiotic or antimicrobial use,?

Secondary research questions included:

2. What are the barriers and facilitators to maintaining these information relationships?
3. What are the mechanics of these relationships?
4. What are the interventions and recommendations to promote positive relationships?
5. What is the impact on rational antimicrobial use?

### Literature search

We searched NCBI PubMed, CAB Abstracts, ProQuest Social Science Premium Collection, and APA PsycINFO for primary peer-reviewed studies published from 2000 to April 10^th^ 2025, in any language. 2000 was chosen to represent the past twenty-six years of literature to maintain feasibility and relevance. Grey literature, opinion pieces, editorials, commentaries, and review papers were excluded. Search strings were iteratively developed with a librarian to optimize precision and relevance based on the primary research question. Search strings are published within the OSF Protocol [7] and in Supplementary Material S2. While our focus was on antibiotic use, our search included literature on antimicrobial use. As such, we will use the encompassing term antimicrobial hereafter. Since we identified a large body of literature, reference lists of included studies and expert recommendations were not hand-searched to identify additional relevant studies.

### Inclusion and exclusion criteria and screening

Inclusion and exclusion criteria are provided below in Table 1.

**Table 1:** Inclusion and exclusion criteria

	Inclusion	Exclusion
Population	Farm or farm workers (those that work with production animals), from all types of farms and any geography.	Agricultural workers working with crops, wildlife or companion animals. Studies that focus on veterinarians as the population of interest.
Outcome	Studies that provided information addressing the primary research question of whom or where workers get information or advice about antibiotic or antimicrobial use in production animals. We defined production animals as any animal raised to produce products for consumption including avian, swine, bovine, caprine, camel, equine, rabbit, ovine, fish, bees, mollusks, mink, ferrets, or crustacean species.	Information solely on where antibiotics are purchased without explicit mention of advice or exchange of information and studies that focused on intervention evaluations. Information related to advice broadly on animal welfare or biosecurity practices (i.e. not specific to antimicrobials).
Study Design	Qualitative or quantitative primary peer-reviewed studies (all study designs) in any language from 2000 onwards	Grey literature, book and book chapters, opinion pieces, editorials, commentaries, review papers

All records were imported into Covidence [8], and duplicates were removed. Two reviewers independently screened titles and abstracts for relevance using the inclusion criteria provided in Table 1, resolving conflicts through discussion. Full texts of studies retrieved after abstract screening were assessed for eligibility by two reviewers to ensure that they met the inclusion criteria defined in Table 1. Studies focusing on veterinarians as the population of interest were excluded during full text review.

### Data charting and analysis

Data charting was performed using structured Excel forms to capture study meta-data (authors, country, year, study design, sample size, and farm type), points of influence, communication pathways, barriers and facilitators, and explicitly stated impacts on antimicrobial use or recommendations. One researcher inductively extracted codes for points of influence, barriers and facilitators for each study. The number of individual codes were listed, and thematically similar codes were amalgamated and defined based on iterative discussions between two researchers (Tables 2, 3, 4). For secondary questions 3–5, relevant quotes were extracted into the Excel form.

**Table 2:** Definitions for points of influence

Points of Influence	Definitions
**DIRECT**	**Direct influences are those with direct and frequent contact with farmers**
Advisors	External individuals that provide advice to farmers in some formal or informal capacity, but are not healthcare workers or veterinarians
Animal health workers	Professionals who provide care and support for animals, not explicitly addressed as a veterinarian in study
Family/friends	Family or friends (including neighbors) of farmer or farm worker
Local service providers	Local providers, organizations and businesses that support agricultural activities
Paraprofessionals	An individual that provides professional animal services without being fully licensed to independently practice
Peers	Peers, or other farmers
Retail sellers	Supply stores for farmers selling drugs and feed, including agrovets
Veterinarian	Licensed veterinarians that treat animals, including both government and private veterinarians
**INTERMEDIARY**	**Intermediate influences represent organizational or market-level actors that shape farmers/producers antimicrobial use practices or disease management practices**
Animal dealers	Supply farms with animals to raise
Community	External community in which farm exists
Consumers	Individuals that purchase products
Cooperatives/organizations	Farmer cooperative or organizations that work together
Distributor (Intermediary)	An intermediary person or business that distributes animal feed to retail seller
Non-governmental organization (NGO)	Non-governmental organization
Processing companies/buyers	Individuals or businesses that buy raw products for downstream processing, including feed mills
Product vendors	Individuals or businesses selling finished products (i.e. milk vendor)
School and continued education	Any form or school or organized continued education
**DISTAL**	**Distal influences act at the structural or policy level, often shaping incentives, regulations, or educational guidance**
Government	All forms of persons, offices or laws from the government (excluding government veterinarians)
Industry bodies	Organizations that represent and advocate for the interests of a specific industry or sector
Pharmaceutical company	Belonging to a drug or pharmaceutical company
Private sector	Businesses owned and operated by private individuals or groups, distinct from government or public entities
Researchers	Academic researchers and published academic research
**CROSS-CUTTING**	**Cross-cutting influences refer to individuals, groups, or mechanisms that influence decision-making about antimicrobial use across the entire farming system**
Internet	Advice and information specifically hosted and consumed on the internet, excluding social media
Mass media	Forms of media excluding internet
Social media	Online personal experiences and feedback on social media and forums

**Table 3:** Definitions for barriers

Barrier in utilizing information source	Definition
Absence of diagnostic facilities	Absence of diagnostic facilities or equipment at source
Access/availability	Lack of access to services, including poor reliability and issues with seeking service due to proximity
Cost	Financial constraints and cost of services
Increased workload	Seeking out advice/information increases workload or takes too much time
Lack of competency/knowledge	Perceived lack of competency of information source
Lack of trust	Perceived lack of trust in relationship, including perceived biases of information source, especially due to outside financial interests
Language barriers	Difficulties in communicating due to differences in language
Limited enforcement	Limited enforcement and monitoring mean no need to seek out advice
Magnitude of problem	Farmers do not seek out information source for diseases or problem not perceived as severe

**Table 4:** Definitions for facilitators

Facilitator in utilizing information source	Definition
Approachability	Service/advice is perceived as approachable
Access/availability	Service/advice is readily available and/or good access to service/advice.
Financial incentives	Financial incentives for services
Good communication	Good mechanisms of communication by source
Good relationship/existing relationship	Good and/or existing relationships between source and farmer
Magnitude of problem	A severe disease or problem will drive farmers to seek information source
Number of animals	Seeking out information sources depends on the numbers of animals
Peer pressure	Seeking out information sources is influenced by peer pressure
Specialty/competence	Experience and specialty knowledge is a positive factor to seek out information source
Trust (positive)	High levels of trust between source and farmer

Points of influence were defined as whom or where (i.e. individuals, groups or communication pathways) workers get information or advice about antibiotic use in production animals or who influences antibiotic use in production animals. Two authors examined how actors interacted with farmers, and points of influence were ultimately categorized by their directness of interaction with producers and farmers specifically as direct, intermediary, distal or cross-cutting (Table 2). Countries were classified by World Health Organization (WHO) regions for analysis: African Region (AFRO), Region of the Americas (AMRO), Eastern Mediterranean Region (EMRO), European Region (EURO), South-East Asian Region (SEARO), and Western Pacific Region (WPRO). The number of times a point of influence, barrier and facilitator were mentioned were numerated and a qualitative narrative analysis was performed on mechanics, impact of advice on antibiotic use and recommendations. For the qualitative analysis, relevant quotes that were extracted during data charting were compiled in a word processing document for analysis and synthesis. Recommendations were coded for themes and those that represented the same theme were then compiled. For impact on rational use, the specific impact was noted during synthesis and grouped as such.

## Results

**Fig. 1:**
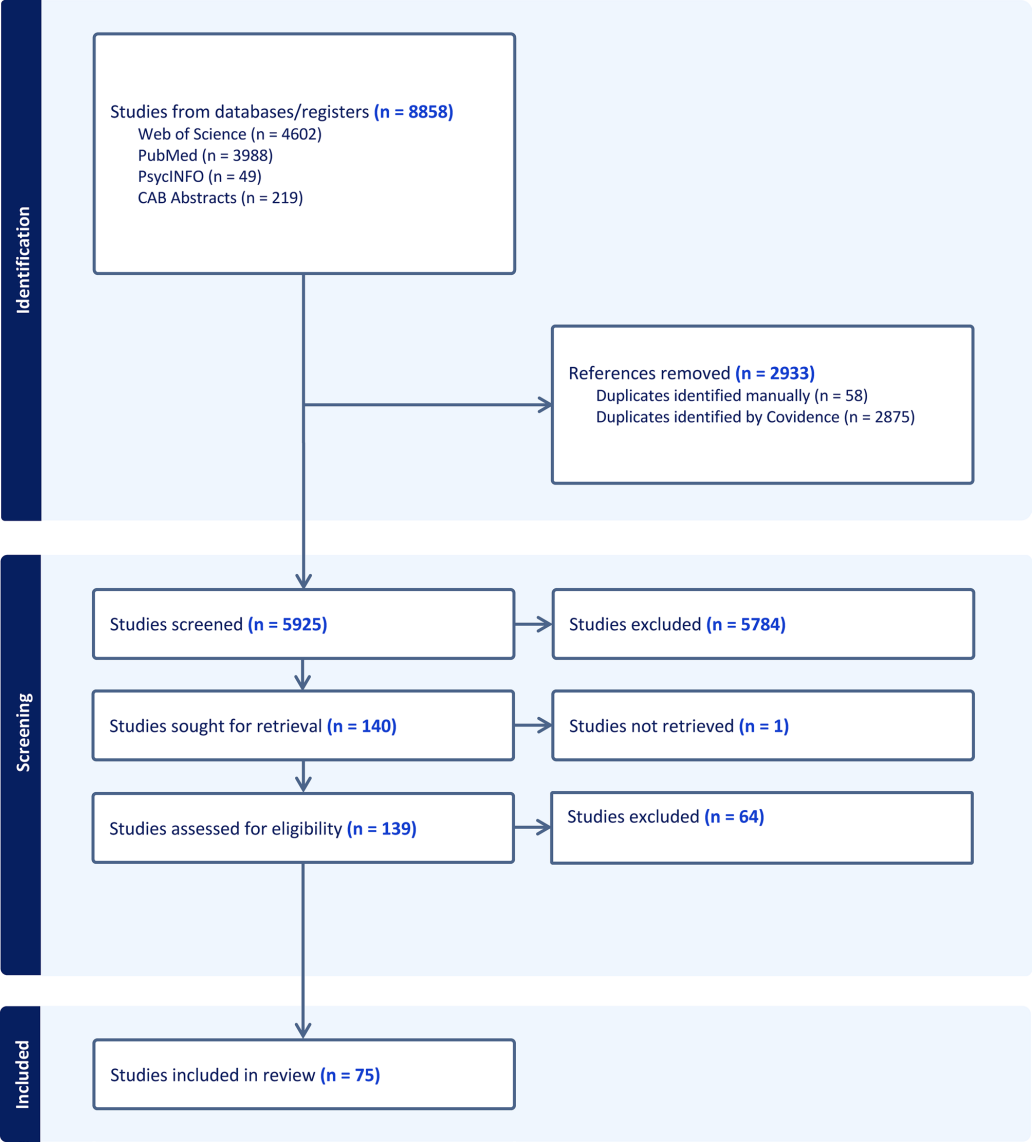
PRISMA flowchart generated from Covidence

Our search retrieved 5925 records after removal of duplicates (Fig. 1). After abstract review of each search by two reviewers, 140 studies were moved to full text retrieval for assessment of eligibility (Table 1). After review of full texts, 64 did not meet inclusion criteria, and one text could not be retrieved. Thus, a total of 75 papers were included for data charting.

### Characteristics of studies

**Fig. 2:**
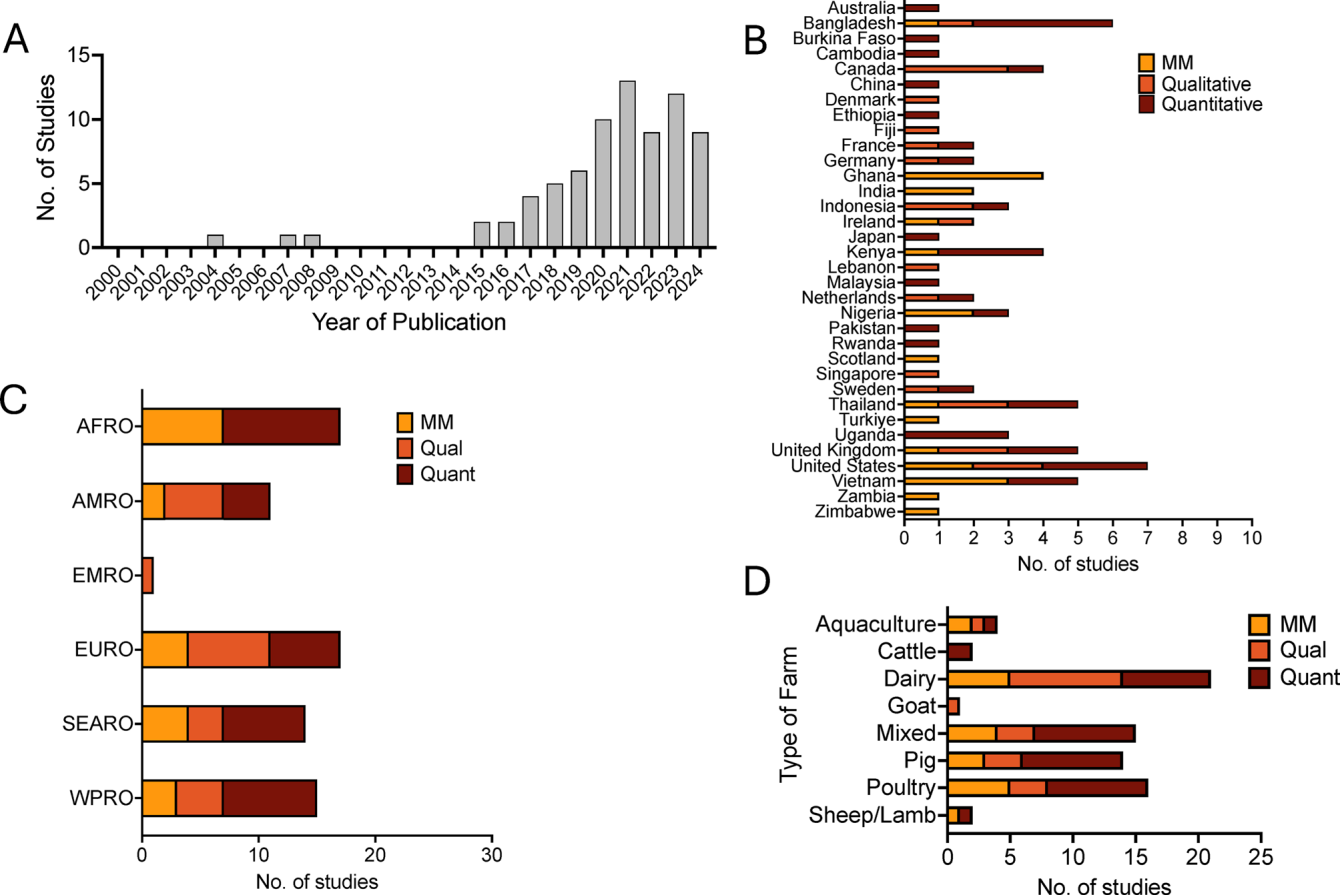
**A**. Distribution of studies included by year of publication **B**. Distribution of studies by country in which study takes place, broken down by study type. **C**. Distribution of studies by WHO Region in which study takes place, broken down by study type. **D**. Distribution of studies by type of farm study, broken down by study type. MM = mixed methods (*N* = 20), qual = qualitative (*N* = 35), Quant = quantitative (*N* = 20). AFRO-African Region, AMRO – Region of the Americas, EMRO – Eastern Mediterranean Region, EURO- European Region, SEARO- South-East Asian Region, WPRO – western Pacific Region

Supplementary Material S3, and a summary of papers included are provided in Tables 5, 6, 7. Of the 75 studies, publication dates ranged from 2004 to 2024, with the most studies for a year published in 2021 (13/75, 17.3%) and 70.7% (53/75) published from 2020 onwards (Fig. 2A). Studies took place in 28 countries broken down by WHO region as follows: African Region (AFRO (17/75, 22.7%)), Region of the Americas (AMRO (11/75, 14.7%)), Eastern Mediterranean Region (EMRO (1/75, 1.3%)), European Region (EURO (17/75, 22.7%)), South-East Asian Region (SEARO (14/75, 18.7%)) and Western Pacific Region ((WPR (15/75, 20%)) (Fig. 2B). Only one study was from the Eastern Mediterranean Region and there were no qualitative studies from the African region. Among specific countries, the highest number of studies were from the United States (7), followed by Bangladesh (6), Vietnam (5), United Kingdom (5) and Thailand (5) (Fig. 2C). For methodology of the 75 studies: 26.7% (20/75) were mixed-methods studies, 26.7% (20/75) were qualitative studies and 46.7% (35/75) were quantitative studies. For farm type, most studies focused on dairy farms (28%, 21/75), followed by poultry farms (21.3%, 16/75) (Fig. 2D).

**Table 5:** Mixed methods studies

Authors	Year Published	Country of Study	Type of Farm	Points of Influence	Barriers	Facilitators
Adebowale, O. O. et al.[9]	2020	Nigeria	Pig	Internet, Peers, Veterinarian, Government	Access/availability, Cost	Magnitude of problem
Afakye, K. et al.[10]	2020	Ghana, Kenya	Poultry Layer	Retail sellers, Community, Peers, Veterinarian, Distributor (Intermediary)	Access/availability, Absence of diagnostic facilities	None described
Borelli, E. et al.[11]	2023	Scotland	Dairy	Veterinarian, Internet, Processing companies/buyers, Mass media	None described	None described
Caudell, M. A et al.[12]	2020	Zambia, Zimbabwe	Broiler	Veterinarian, Animal health workers, Friends/family, Retail sellers	Access/availability, Lack of trust, Lack of competency/knowledge	None described
Dandi, S. O. et al.[13]	2024	Ghana	Cage Fish	Family/friends, Retail sellers, Veterinarian	Access/availability, Absence of diagnostic facilities	None described
Dhayal, V. S.et al.[14]	2023	India	Buffalo, Cattle, Goat, Sheep, Poultry	Doctor, Peers, Retail sellers, Mass media, Pharmaceutical company	Cost	Trust (positive)
Diana, A.et al.[15]	2021	Ireland	Pig	Peers, Veterinarian, Advisors, Researchers	Access/availability, Lack of trust, Magnitude of problem	Magnitude of problem
Doidge, C. et al.[16]	2021	UK	Lambs	Veterinarian, Social media, Peers	None described	None described
Friedman, D. et al.[17]	2007	USA	Dairy	Veterinarian, Pharmaceutical company, Peers, Animal health workers	Language barriers, Cost, Time Constraints, Lack of trust	None described
Lekagul, A et al.[18]	2020	Thailand	Pig	Family/friends, Peers, Veterinarian, Pharmaceutical company, Processing companies/buyers	None described	None described
Luu, Q. H. et al.[19]	2021	Vietnam	Chicken, pig	Veterinarian, Pharmaceutical company, Processing companies/buyers, Retail Sellers, Family/friends	Limited enforcement, Absence of diagnostic facilities	None described
Moore, D. A et al.[20]	2021	USA	Dairy, calf	Veterinarian, Peers	None described	Good communication
Nuvey, F. S. et al.[21]	2023	Ghana	Ruminant (sheep, goat and cattle farmers)	Veterinarian, Retail sellers	Cost, Access/availability	Magnitude of problem, Number of animals
Ojo, O. E.et al.[22]	2016	Nigeria	Chicken, sheep, goat, turkey, pigs and cattle	Veterinarian	None described	None described
Ozdikmenli Tepeli, S.[23]	2023	Turkiye	Dairy	Veterinarian	None described	None described
Patnaik, N. et al.[24]	2020	India	Dairy	Veterinarian, Retail sellers, Product vendors, Cooperative/organizations	Cost	Trust (positive), Access/a vailability
Tasmim, S. T et al.[25]	2023	Bangladesh	Poultry	Animal dealers, Retail sellers, Veterinarian, Peers, Mass media, School and continued education	Limited enforcement, Access/availability	None described
Tran Thi K. C. et al.[26]	2017	Vietnam	Aquaculture	Peers, Retail sellers	Access/availability	None described
Truong, D. B. et al.[27]	2019	Vietnam	Chicken, duck	Veterinarian, Animal health workers	None described	Trust (positive)
Turkson, P. K.[28]	2008	Ghana	Poultry	Veterinarian, Paraprofessionals, Retail sellers, Peers	Cost	None described

**Table 6:** Qualitative studies

Authors	Year Published	Country of Study	Type of Farm	Points of Influence	Barriers	Facilitators
Adam, C. J. M. et al.[29]	2020	France	Poultry	Veterinarian, Mass media, Cooperatives/organizations, Consumers, School and continued education, Peers	None described	Good relationship/existing relationship, Trust (positive), Access/availability
Bradford, H. et al.[30]	2022	Northern Ireland and England	Pig	Veterinarian	None described	Trust (positive), Good relationship/existing relationship
Cobo-Angel, C. et al.[31]	2021	Canada	Dairy	Veterinarian, Family/friends, Peers, Internet, Social media	Lack of competency/knowledge	None described
Dankar, I. et al.[32]	2022	Lebanon	Dairy	Veterinarian	Cost, Limited enforcement	Good relationship/existing relationship
de Jong, E et al.[33]	2024	Canada	Dairy	Veterinarian	Access/availability	Approachability
Ekakoro, J. E. et al.[34]	2019	USA	Beef Cattle	Veterinarian, Peers, Cooperatives/organizations, Governments, Mass media	Access/availability	Good relationship/existing relationship, Access/availability, Magnitude of problem
Fischer, K. et al.[34]	2019	Sweden	Dairy	Veterinarian	Cost	Trust (positive), Access/availability, Specialty/competence, Magnitude of problem
Hibbard, R. et al.[35]	2023	Indonesia	Poultry	Private sector, Peers, Cooperatives/organizations	Absence of diagnostic facilities, Lack of trust	Access/Availability
Huey, S. et al.[36]	2021	Ireland	Dairy	Peers, Veterinarian	Access/availability, Lack of competency/knowledge	Peer pressure
Ida, J. A. et al.[37]	2023	Canada	Dairy	Veterinarian	None described	Trust (positive)
Jannah, N. et al.[38]	2024	Indonesia	Ruminant	Paraprofessionals, Veterinarian, Peers, Family/friends	Access/availability	
Khan, X. et al.[39]	2022	Fiji	Livestock	Cooperative/Organizations, Peers, Social media	Access/availability, Lack of trust, Lack of c ompetency/ knowledge	None described
Landfried, K.L. et al. [40]	2018	USA	Goat	Veterinarian, Social Media, Peers	Cost, Lack of competency/knowledge	Trust (positive), Access/availability, Magnitude of problem
Lekagul, A. et al.[41]	2021	Thailand	Pig	Veterinarian, Pharmaceutical company, Researchers		Financial incentives
Lim, J. M. et al.[42]	2020	Singapore	Aquaculture	Peers, Government	None described	None described
Masud, A. A. et al.[43]	2020	Bangladesh	Broiler Poultry	Animal Dealers	Cost	Access/Availability
Pate, L. A. et al.[44]	2023	UK	Dairy	Veterinarian, Peers, Mass media	Absence of diagnostic facilities	Peer pressure
Skjølstrup, N. et al.[45]	2021	Denmark	Dairy	Veterinarian, Government, Consumers, Community, Mass media, Peers	Cost	None described
Swinkels, J. M. et al.[46]	2015	Netherlands and Germany	Dairy	Veterinarian, Animal health workers, Peers, Mass media, Government	Access/availability, Lack of trust	None described
Thongyuan, S. et al.[47]	2024	Thailand	Pig	Veterinarian	Increased workload	None described

**Table 7:** Quantitative studies

Authors	Year Published	Country of Study	Type of Farm	Points of Influence	Barriers	Facilitators
Adam, C. J. M. et al.[48]	2019	France	Broiler	Paraprofessionals		
Aniume, T et al.[49]	2023	United States (Tennessee & Georgia)	Goat and sheep	Peers, Internet, Veterinarian		
Backhans, A. et al.[50]	2016	Sweden	Pig	Veterinarian		
Chowdhury, S. et al.[51]	2022	Bangladesh	Poultry	Veterinarian, Retail Sellers, Paraprofessionals, Pharmaceutical company, Feed distributer (intermediary)		
Chowdhury, S. et al.[52]	2022	Bangladesh	Fish	Retail Sellers, Peers, Local service providers		
Cobo-Angel, C. et al.[53]	2022	Canada	Dairy	Veterinarian	Access/availability, Cost	None described
Coyne, L. et al. [54]	2020	Indonesia	Broiler	Retail Sellers, Veterinarian, Paraprofessionals, Peers	Access/availability	None described
Doidge, C. et al.[55]	2021	UK	Sheep	Veterinarian	None described	None described
Doyle, E. et al.[56]	2022	Australia	Dairy, Cattle	Veterinarian, Government, Peers, Industry bodies, Internet, Social media, Processing companies/buyers	None described	None described
Farhan, M. et al.[57]	2024	Pakistan	Dairy	Veterinarian, Peers, Family/friends	Cost, Lack of trust	None described
Geta, K. and Kibret, M.[58]	2021	Ethiopia	Poultry, beef and dairy	Veterinarian	Cost	None described
Hassan, M. M. et al.[59]	2021	Bangladesh	Poultry (Broiler and layer)	Veterinarian	Cost	None described
Hirwa, E. M. et al.[60]	2024	Rwanda	Cattle	Veterinarian, Family/friends, Mass media, School and continued education	None described	None described
Isomura, R. et al.[61]	2017	Japan	Pig	Veterinarian	None described	None described
Jones, P. J. et al.[62]	2015	England and Wales	Dairy	Veterinarian, Consumers, Family/friends, Peers, Cooperative/organizations, Processing company/buyers	None described	None described
Kemp, S. A. et al.[63]	2021	Kenya	Mixed crop–livestock	Veterinarian, Retail sellers	None described	None described
Kigozi, M. M. and Higenyi, J.[64]	2017	Uganda	Layer poultry	Animal Health Workers, Retail sellers, School and continued education	Lack of competency/knowledge, Cost	
Kisoo, L. et al.[65]	2023	Kenya	Cattle	Veterinarian, Paraprofessionals	None described	None described
Nohrborg, S. et al.[66]	2024	Vietnam	Chicken	Veterinarian, Peers, Retail sellers, Processing companies/buyers, Family/friends	Access/availability	None described
Nohrborg, S. et al.[67]	2022	Uganda	Pig	Veterinarian, Retail Sellers, Animal health workers, Peers	None described	None described
Nuangmek, A. et al.	2018	Thailand	Poultry, pig	Veterinarian	None described	None described
Okello, D. M. et al.[68]	2022	Uganda	Pig	Veterinarian, Mass media, Cooperatives/organizations, Government, NGO	None described	None described
Omolo, J. et al. [69]	2024	Kenya	All livestock	Retail Sellers, Veterinarian	Access/availability	
Oyebanji, B. O and Oyebisi, M. O.[70]	2017	Nigeria	Cattle, Fish, Poultry	Peers, Veterinarian	None described	Access/availability
Pham-Duc, P. et al.[71]	2019	Vietnam	Pig, poultry and aquaculture	Mass media, School and continued education	None described	None described
Rahman, Md S. et al.[72]	2021	Bangladesh	Dairy	Veterinarian, Retail sellers, Peers	None described	None described
Ratanapob, N. et al. [73]	2024	Thailand	Dairy	Veterinarian, Peers, Retail sellers, School and continued education, Government, Internet, Mass media, Cooperatives/organizations	None described	Trust (positive)
Sadiq, M. B. et al.[74]	2018	Malaysia	Ruminant (mixed and single)	Veterinarian	None described	None described
Sawadogo, A. et al.[75]	2023	Burkina Faso	Poultry	Veterinarian, Animal health workers	None described	None described
Schneider, S. et al.[76]	2018	Germany	Pig	Veterinarian, Researchers, School and continued education, Peers, Social media	None described	None described
Si, R. et al.[77]	2023	China	Hog/pig	Internet	None described	Good communication
Ström, G. et al.[78]	2018	Cambodia	Pig	Veterinarian, Retail sellers	None described	None described
van Asseldonk, M. et al.[79]	2020	Netherlands	Pig	Veterinarian, Retail sellers	None described	None described
Vasquez, A. K. et al.[80]	2019	USA	Dairy	Veterinarian, Peers, Processing companies/buyers	Cost, Increased workload	Good relationship/existing relationship, Trust (positive)
Zwald, A. G. et al.[81]	2004	USA	Dairy	Veterinarian, Pharmaceutical company, Peers	None described	None described

### Points of influence

**Fig. 3:**
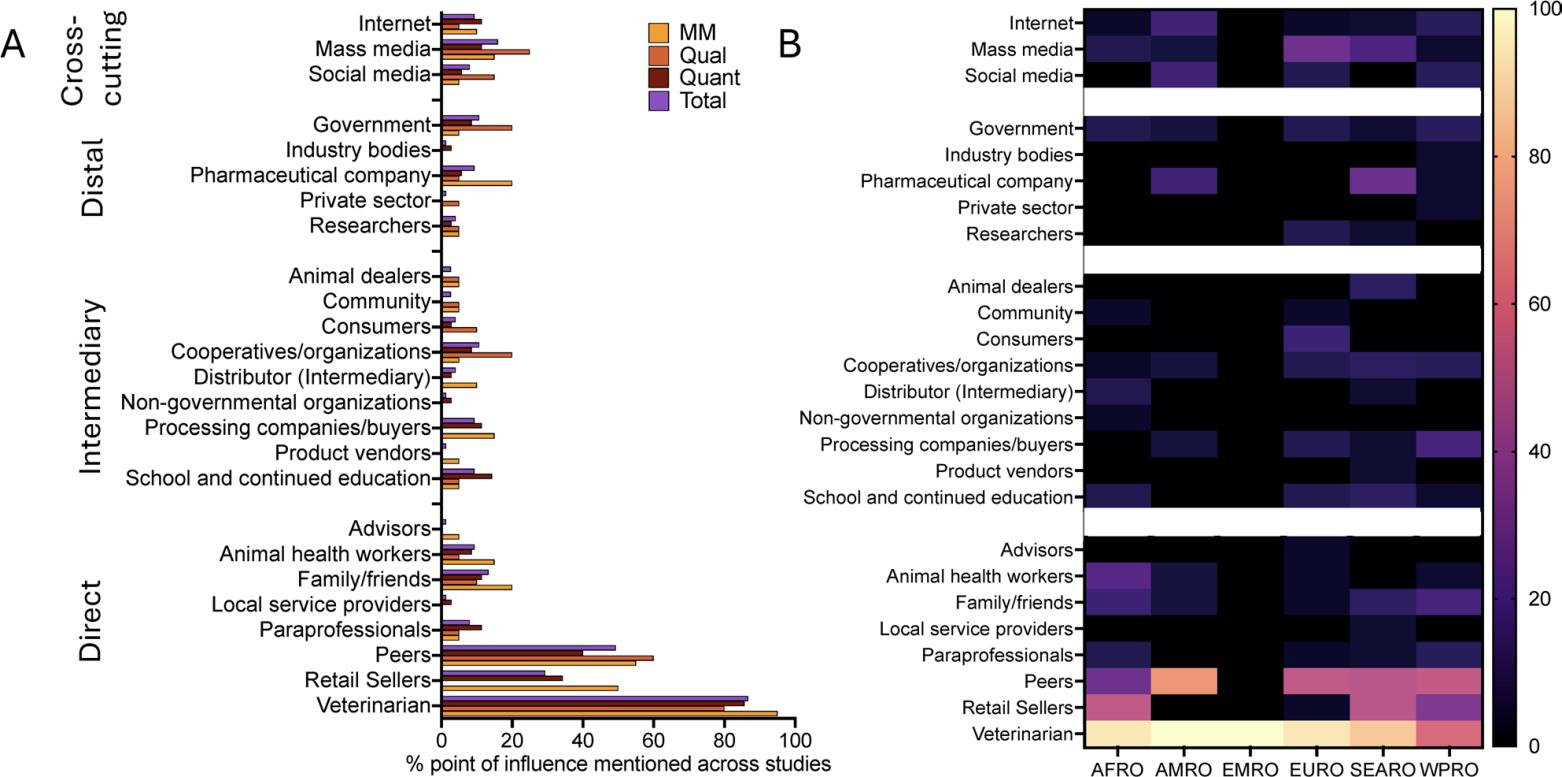
**A**. Percentage mention of point of influence across studies, by study type and across all studies included **B**. Heatmap showing the percentage mention of point of influence by number of total studies within a specific WHO region AFRO-African Region (*N* = 17), AMRO – Region of the Americas (*N* = 11), EMRO – Eastern Mediterranean Region (*N* = 1), EURO- European Region (*N* = 17), SEARO- South-East Asian Region (*N* = 14), WPRO – western Pacific Region (*N* = 15). Points of influence are sorted by directness of interaction. MM = mixed methods (*N* = 20), Qual = qualitative (*N* = 35), Quant = quantitative (*N* = 20), Total = all study types (*N* = 75)

Twenty-five points of influence were noted (Table 2). Across all studies (*N* = 75), veterinarians were the most mentioned points of influence (65/75, 86.7%), followed by peers at 49.3% (37/75) and retail sellers at 29.3% (22/75) (Fig. 3A). Across all coded points of influence mentioned (*N* = 228), veterinarians, peers and retail sellers represented 28.5% (65/228), 16.2% (37/228) and 9.6% (22/228) of total instances (Fig. S1). Qualitative studies, which are rooted in more free-form answers, noted higher percentages for cooperatives/organizations, mass media, and social media compared to quantitative and mixed-methods studies, which often had more defined answers (Fig. 3A). Analyzing influencers by WHO Region (Fig. 3B), we note some regional differences. For example, studies from AMRO did not mention retail sellers as points of influence, while studies from AFRO and SEARO did not mention social media. Since quantitative questions were posed slightly differently, we could not determine cumulative statistics on usage.

**Fig. 4:**
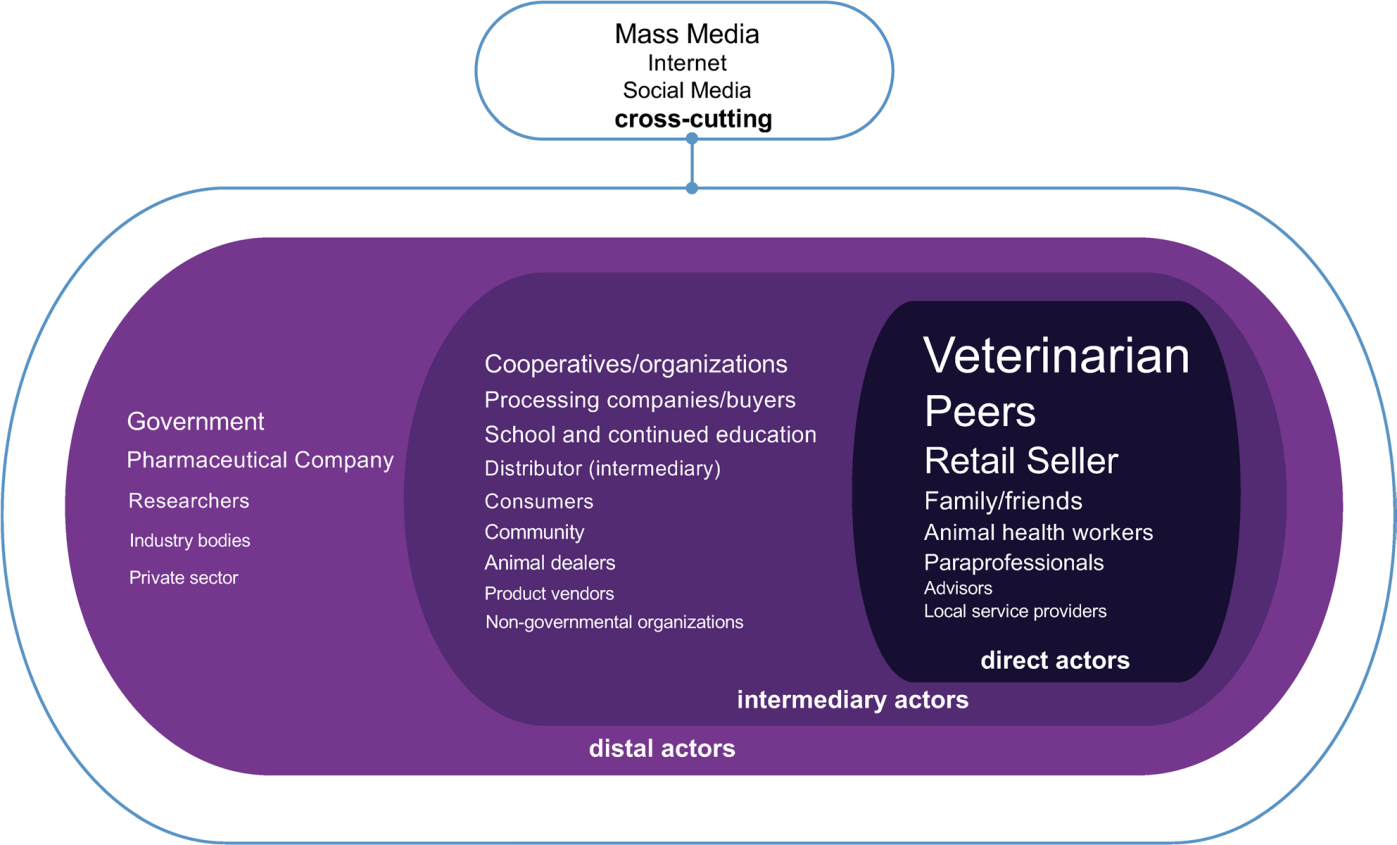
Map of actors influencing antimicrobial use by farm workers. Size of font is proportional to usage identified in scoping review

Mapping the points of influence illustrated how the farming system is organized into direct actors, intermediary actors, distal actors and cross-cutting influences (Fig. 4, Table 2). Notably, the most frequently mentioned points of influence were direct actors (veterinarians, peers, retail sellers), though actors at all levels exerted influence.

### Barriers & facilitators

**Fig. 5:**
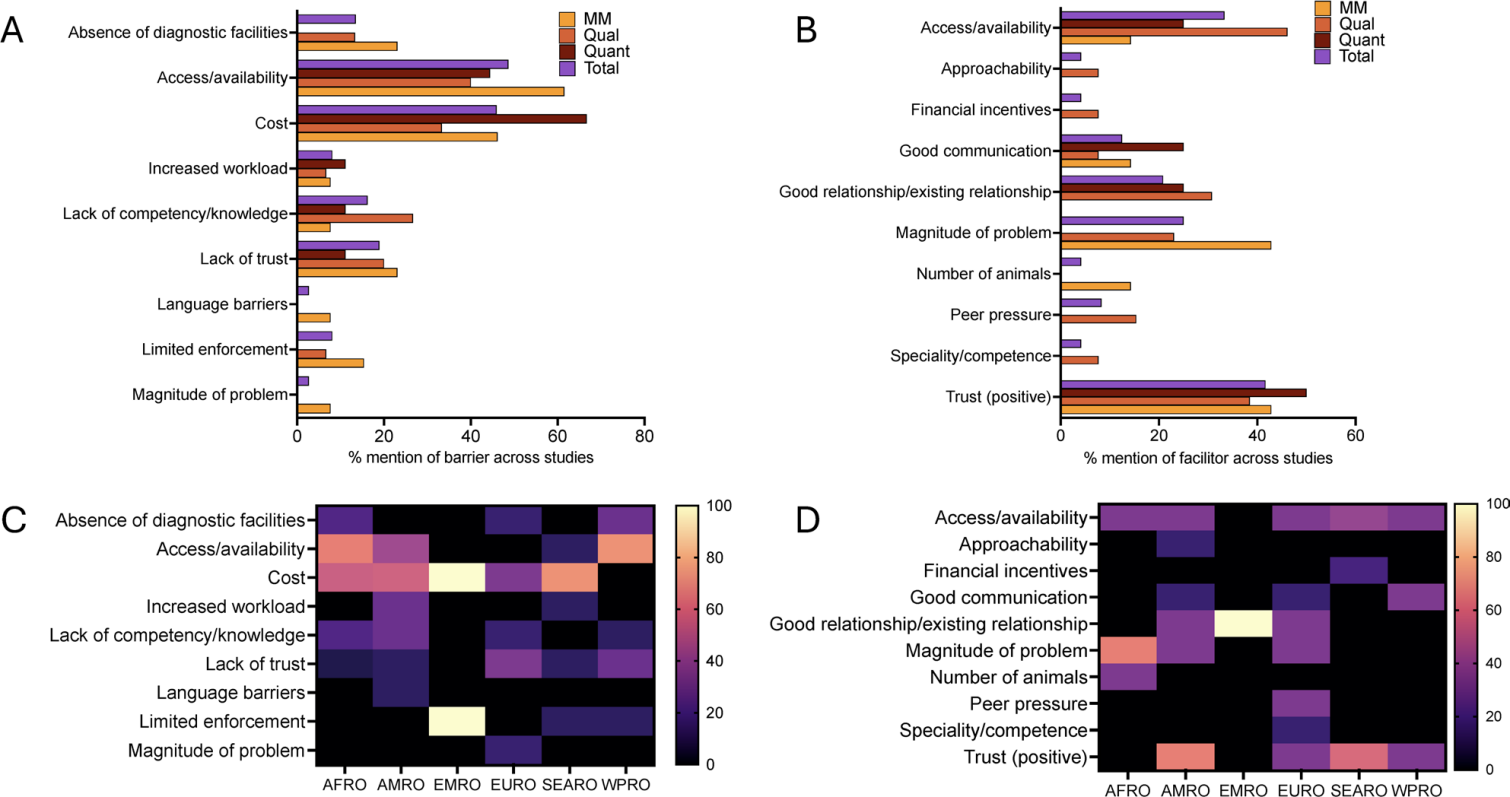
**A**. Percentage mention of barrier across all relevant studies (those that reported barriers) included, by study type (MM- *N* = 13, qual- *N*=,15 Quant- *N* = 9) and as a percentage of total studies (*N* = 37) **B**. Percentage mention of facilitator across all relevant studies (those that reported facilitators), by study type (MM- *N* = 7, qual- *N* = 13, Quant- *N* = 4) and as percentage of the total studies (*N* = 24) **C**. Heatmap showing percentage barriers mentioned among total studies by WHO region (AFRO- *N* = 9, AMRO- *N* = 7, EMRO-*N* = 1, EURO-*N* = 6, SEARO-*N* = 7, WPRO-*N* = 7) **D**. Heatmap showing percentage facilitators among total studies by WHO region(AFRO- *N* = 3, AMRO- *N* = 6, EMRO-*N* = 1, EURO-*N* = 6, SEARO-*N* = 5, WPRO-*N* = 3). AFRO-African Region. AMRO – Region of the Americas, EMRO – Eastern Mediterranean Region, EURO- European Region, SEARO- South-East Asian Region, WPRO – western Pacific Region. MM = mixed methods, Qual = qualitative, Quant = quantitative, Total = all study types

Data for barriers and facilitators for utilizing points of influence for information were also inductively coded and grouped. Nine barriers and ten facilitators emerged from our analyses (Fig. 5, Table 3,4). Out of all relevant studies (those that reported barriers (*N* = 37) or facilitators (*N* = 24)), the most frequent barriers were access/availability (48.6%, 18/37) and cost (45.9%, 17/37), followed by lack of trust (18.9%, 7/37) (Fig. 5C). The most frequent facilitator was trust (41.7%, 10/24), followed by access/availability (33.3%, 8/24) and magnitude of problem (25.0%, 6/24) (Fig. 5B). Percentages across all coded barriers and facilitators mentioned are presented in Fig. S2. Again, qualitative studies noted increased workload while the other study types did not (Fig. 5A). Analyzing barriers and facilitators by WHO Region (Fig. 5C&D), we note that access/availability is a more frequent barrier (>50%) among studies in AFRO, and WPRO. Cost was noted as a major barrier in all regions, except for WPRO.

#### Impact of advice and AMR and antibiotic use

Twelve studies [9, 10, 15, 18, 36, 48, 54–56, 65, 68, 75] explicitly noted or probed the relationship between advice and antibiotic use. From those that did, the relationships were either neutral (no difference, 2 studies [54, 55]), positive (facilitating appropriate antibiotic use, 3 studies [9, 15, 36] (2 veterinarian advice and 1 peer influence)) or negative (negative influence on prudent use, 1 study [75], advice from veterinarian/professional). One study highlighted different impacts based on the source of advice, specifically, advice from agrovets and veterinarians was correlated with a higher likelihood of an antibiotic prescription, while advice from community/extension animal health workers was more likely to result in producers consuming animal products during withdrawal periods [10]. Five studies noted an association of advice with a higher probability of antibiotic use but did not explicitly clarify if this use was appropriate (5 studies [18, 48, 56, 65, 68]). For example, one study highlighted the association with certain information sources (radio, farmer organizations) and self-administering antibiotics [68].

## *Mechanicsof relationships*

As part of the review we also assessed mechanics of the relationships or how the relationship functions (i.e. interactions) between the farmer and the point of influence. One study from Swedish dairy farms noted that a farmer had a deal with their veterinarian to allow antibiotics at home for quick access during the weekend, and another farmer inducated veterinarian sanctioned small-scale hoarding of antibiotics [34]. Two studies from poultry farms in Bangladesh highlighted that financial arrangements obligated some farmers to rely on advice and information from feed dealers [43, 51]. Another study from Thailand on pig farming noted that pharmaceutical companies influenced commercial farms by offering discounts and gifts [41]. Reflecting on the barriers and facilitators previously discussed, many farmers across regions only consulted veterinarians in severe cases [35, 40]. Often, advice was provided without diagnostics or site visits (i.e. through phone calls or text messages), noted in studies from Lebanon and Canada [31, 32]. Given the number of respondents who used retail shops as sources for advice, there is a threat of informal advice from drug sellers who are not properly trained [28]. Peers were often viewed as more accessible than veterinarians [82]. One study also noted that the most effective consults were those when both parties contributed their ideas [36].

### Recommendations

Among recommendations identified by studies included within our scoping review, a few themes emerged:

#### Improve communication and trust

Reflective of the barriers and facilitators was the suggestion to improve relationships, communication and trust between farmers and points of influence, largely veterinarians [14, 38, 40, 56, 80]. However, specifics on how to achieve this were limited. To improve communication, one study found a desire for treatment information written in plain and simple language, in formats including *“laminated posters and flowcharts for the barn, videos, and educational seminars*” [17]. Communication by a positive or credible group can improve confidence in good antimicrobial usage practices [46, 80].

Another common suggestion was to increase awareness, such as through media campaigns [9, 57]. Including veterinarians in AMR awareness could be effective as they are seen as a trusted source [24]. Notably, there may be a digital divide, and strategies to both close this gap in technological literacy and provide information through both electronic communication and non-electronic avenues (such as hard-copy brochures through the mail) are suggested [62, 77]. One study found that individual advice was the preferred way to gather knowledge [79].

#### Increase training and education

One of the most common recommendations was to improve the training and education of both farmers and stakeholders who influence farmers [24, 28, 40, 49, 64, 72, 75, 78]. This included increased training programs by veterinarians and veterinary authorities for farmers [20, 27], as well as inclusive programs that deliver practical guidance to empower farmers [49]. Renumerated seminars might also increase participation [80].

#### Improve affordability and availability

Even though access/availability was the most mentioned barrier, surprisingly few studies addressed this in the recommendations. While some mentioned making services more available, affordable [9, 75] and increasing funding for veterinary services [21], there was little discussion of the mechanisms that could be used to do this.

#### Empower farmers and consider context

A few studies highlighted the importance of considering the structural, social and economic networks in which farmers exist in [29, 43, 49]. One study suggested that co-creation of knowledge between farmers and stakeholders can support self-responsibility and behavioral change [80]. Another study found that interventions may have to be tailored to different target groups. For example, experienced farmers with limited access require more guidance on antibiotic treatment, whereas young to middle-aged, small-scale farmers with lower levels of education need more support on disease prevention practices [66]. Another study noted that recommendations will likely be considered by farmers with relation to their personal experience, emotions, and cultural context. As such, while advice provided by experts may useful and understood, it still may not ultimately lead to behavioral change [37].

## Discussion

The primary research question of this scoping review was to identify points of influence for information and advice on antimicrobial use for farmers across the farming system. Overall, we observe a complex network of stakeholders, all of whom can exert influence. Each stakeholder has a responsibility and role in appropriate antimicrobial use, and any weak point (or action in own interests) [83, 84] could lead to inappropriate use being perpetuated. This is especially relevant given the circular nature of having peers be a major source of information (i.e. peers or other farmers are also getting advice from these other sources) [85]. We also identified that the mechanics of relationships, in practice, may be different from best practices that are expected.

We found that nearly half of the included studies were quantitative, while qualitative and mixed-methods studies together comprised just over half of the literature. Quantitative approaches often captured predefined sources of advice, whereas qualitative studies tended to identify a wider range of actors and contextual influences through open-ended responses. While quantitative work provided useful estimates of the relative importance of key actors such as veterinarians or peers, qualitative approaches usually provided a more detailed understanding of the broader social and contextual dynamics that shape how farmers seek and use advice on antimicrobial use.

The major facilitators and barriers to seeking advice were related to access/availability, cost and trust. Another facilitator was having a good relationship; however, what constitutes a good relationship was not clearly defined in the papers we captured, although trust may be assumed to be a large component [86–88]. This remains an outstanding question for further study. Analyzing these barriers and facilitators, cost and access/availability can broadly be grouped as market or supply issues, while trust relates to relationship building. However, we acknowledge that these factors are also interconnected, for example, fair and transparent costs or reduction of prohibitive fees for quality services may increase access and help foster sustained relationships [89, 90]. Moreover, we note that many of the barriers and facilitators conceptually overlap as factors while having opposite framing (i.e. poor access as a barrier and good access as a facilitator). For our analysis, we separated them based on the framing and language used in the study. While they may represent the same underlying factor, it is interesting to note the nuance between barriers and facilitators, in that barriers may serve as the first impediment, while facilitators promote uptake of advice. Below, we discuss potential strategies to address these three major issues:

### How to reduce costs of advisory services?

One strategy to reduce the cost of advisory services, particularly of licensed veterinarians, are pooled or organized group services for farmers and cooperatives. This would reduce travel and time costs for veterinarians or service providers, allowing them to visit multiple farms in the same area at once, with potential cost savings passed on to clients. Given that many farmers receive advice from professional organizations or farmer cooperatives (Figs. 3 and 4), there seems to be a structure already in place for negotiating such types of service. For example, coordinated herd health programs implemented through dairy cooperatives in the Netherlands have demonstrated that shared veterinary advisory services can reduce per-farm costs while improving disease prevention and reducing antibiotic use [91]. Another potential way to reduce overall costs is for the government to subsidize preventative measures and incentivize alternative strategies (such as setting up organic farming), to allow smaller producers to afford advisory services and limit antibiotic use. For example, Thailand has implemented government-supported programs promoting Good Agricultural Practices and biosecurity improvements in livestock production, including financial and technical support for farmers to upgrade housing, hygiene, and disease prevention practices [92]. Other market-based mechanisms, such as loyalty programs for recurring service, can build clientele, establish relationships and ensure more regular advisory services [93, 94]. Licensed telehealth advice can also reduce costs and improve accessibility [95–97], although this may have limits for diagnosis and testing, and may lead to the use of antibiotics in cases when it is not truly needed.

Of note, when observing the network of influence, some advice might be provided for free (especially distal and cross-cutting actors or in the case of retail sellers who are not being specifically paid for their advice), while other advice comes at a cost (such as from paraprofessionals/veterinarians, schools or through continued education). Care must be taken with advice that is possibly unsolicited or unverified, which might align more with the interests of the actor rather than the farmer. For example a study from five African countries found antimicrobial advice provided by drug sellers or feed dealers may prioritize product sales rather than appropriate treatment, which can contribute to inappropriate antibiotic use [12]. Thus, an important question remains in the quality of advice from this diverse set of actors, and how to ensure that appropriate use is being promoted at each point.

### How to increase access/availability?

To increase access and availability, especially in rural farming areas, government or public-private programs to train and support professionals who will work in these areas is a possible solution (rural health workforce programs) [98]. For example, in human healthcare, Canada provides loan forgiveness for doctors and nurses working in underserved rural and remote communities [99]. Similarly, in Uganda, the training of community animal health workers who provide basic veterinary advice and services in underserved rural regions has been subsidized by non-governmental organizations, as well as the government itself [100]. This initiative helps address geographic and financial barriers to accessing professional veterinary care [101]. Improved training of paraprofessionals can also expand the pool of professionals providing quality advice. Licensed telehealth networks can also improve access to care, especially in rural areas [97].

Clearer dissemination and use of antimicrobial treatment guidelines may also help support more appropriate antibiotic use. In many countries, species-specific treatment guidelines and antimicrobial stewardship frameworks, aligned with global recommendations, have been developed to inform decision-making and promote judicious use [3, 102, 103]. These guidelines provide practical reference points for veterinarians and producers when selecting treatments, help clarify what constitutes “indiscriminate” or inappropriate use, and include recommendations on drug choice, dosage, and duration. Increased awareness and accessibility of these guidelines, particularly when translated into practical, easy-to-use formats may also support improved antimicrobial stewardship at the farm level.

### How to build trust/relationships?

Training those who support farmers’ decision-making on antimicrobial use and animal health practices can help strengthen relationships. It is important to approach stewardship as a shared responsibility [29] and facilitate a dialogue in which farmers feel valued and empowered. Strategies may include co-creating plans and practicing active listening and acknowledging that farmers’ lived experiences offer valuable insights. However, building trust has become a larger challenge, and political trust has been broadly declining among the general population [104]. Farmer field schools and training programs can also build relationships while improving knowledge and capacity for improved animal nutrition, housing and breeding practices, which can reduce reliance on antibiotics [105].

Many of these approaches will not be universal, given community and cultural contexts and differences in resources required for government involvement. Indeed, we noticed some regional differences, which highlight the need for tailored interventions and approaches based on region. In Africa and Asia, retail sellers are a major source of advice on antimicrobial use (Fig. 3, Tables 5, 6, 7), and as such, enforcement of regulations and education and training of drug and feed sellers is an important part of the advice network to address.

## Limitations

There are limitations that come with the data included in our review. Results from each study may not necessarily be generalized based on the study area [14, 43, 49]. Additionally, not all studies reported participant demographics in detail, and several did not provide a gender or sex breakdown, which limits our ability to assess whether perspectives on antibiotic use differed across gender groups. Of the studies which reported gender, some may be underrepresenting women’s views [27]. Future research should try to include gender demographics, when safe to do so, recognizing that in some areas asking participants to identify their gender may be dangerous [106]. Differing methods of collection of survey information and interviews among studies, including self-administration, person-to-person and telephone, may impact results and may lead to bias from social pressure towards certain answers (social desirability bias) [44, 73]. Potential recall bias or the validity of self-reported information is also an issue with surveys and interviews [49]. Several studies also used purposive sampling, which may introduce sampling bias [30]. In addition, this review focused on peer-reviewed literature and excluded grey literature, which may contain additional information on stewardship initiatives and capacity-building activities that are not always reported in academic publications. This represents a knowledge base that could be targeted with future research.

## Conclusions

In this scoping review, we identify and map the various points of influence and communication pathways through which farmers and farm workers receive information or advice on antimicrobial use. We also identified major barriers and facilitators and recommended potential strategies to address barriers. Our analysis revealed a complex network of stakeholders shaping antimicrobial practices, highlighting points of influence that are often overlooked in conventional discussions of information flow. However, this also poses a challenge, as efforts to promote knowledge and awareness of appropriate antibiotic use and the consequences of antimicrobial resistance must navigate multiple layers of actors, each with potentially different interests. By identifying the actors most frequently consulted for advice or information according to the context, more targeted interventions can be designed. An important remaining question, which should be addressed in future research, surrounds the quality and accuracy of the advice provided by each actor.

## Electronic supplementary material

Below is the link to the electronic supplementary material.

**Supplementary material 1 :** Supplemental File 1

**Supplementary material 2 :** Table S1 and Figures S1 & S2

**Supplementary material 3 :** Table S2

## Data Availability

Scoping review protocol can be found at https://osf.io/g4deu/overview. All relevant data provided in manuscript.

## Abbreviations

AMR: antimicrobial resistance
AFRO: African Region
AMRO: Region of the Americas
EMRO: Eastern Mediterranean Region
EURO: European Region
NGO: Non-governmental organization
OSF: Open Science Framework
SEARO: South-East Asian Region
WHO: World Health Organization
WPRO: Western Pacific Region

## References

[1] Van Boeckel TP, Brower C, Gilbert M, Grenfell BT, Levin SA, Robinson TP, et al. Global trends in antimicrobial use in food animals. Proc Natl Acad Sci USA. 2015;112(18):5649–54. 10.1073/pnas.1503141112. 25792457 10.1073/pnas.1503141112 PMC4426470

[2] Chambers JA, Crumlish M, Comerford DA, O’Carroll RE. Antimicrobial resistance in humans and animals: rapid review of psychological and behavioral Determinants. Antibiot (Basel). 2020;9(6):285. 10.3390/antibiotics9060285. 10.3390/antibiotics9060285 PMC7345344 32471045

[3] Guenin MJ, Studnitz M, Molia S. Interventions to change antimicrobial use in livestock: a scoping review and an impact pathway analysis of what works, how, for whom and why. Preventative Vet Med. 2023;220:106025. 10.1016/j.prevetmed.2023.106025. 10.1016/j.prevetmed.2023.106025 37776605

[4] Munjita SM, Mumba C. Peer knowledge sharing on social media: investigating antibiotic overuse by poultry farmers in Zambia. PAMJ-OH. 2025;16(6). 10.11604/pamj-oh.2025.16.5.45784.

[5] Malijan GM, Howteerakul N, Ali N, Siri S, Kengganpanich M, Nascimento R, et al. A scoping review of antibiotic use practices and drivers of inappropriate antibiotic use in animal farms in WHO Southeast Asia region. One Health. 2022;15:100412. 10.1016/j.onehlt.2022.100412. 36277092 10.1016/j.onehlt.2022.100412 PMC9582544

[6] Tricco AC, Lillie E, Zarin W, O’Brien KK, Colquhoun H, Levac D, et al. PRISMA extension for scoping Reviews (PRISMA-ScR): checklist and explanation. Ann Intern Med. 2018;169(7):467–73. 10.7326/M18-0850. 30178033 10.7326/M18-0850

[7] Ching C, Emdin F. Scoping review protocol: identifying key relationships and points of influence for farmers regarding antibiotic use in production animals. 2025. 10.17605/OSF.IO/G4DEU). Accessed.

[8] Innovation VH. Covidence systematic review software. https://www.covidence.org/. Nov 13, 2025.

[9] Adebowale OO, Adeyemo FA, Bankole N, Olasoju M, Adesokan HK, Fasanmi O, et al. Farmers’ perceptions and drivers of antimicrobial use and abuse in commercial pig production, Ogun state, Nigeria. IJERPH. 2020;17(10):3579. 10.3390/ijerph17103579. 32443741 10.3390/ijerph17103579 PMC7277550

[10] Afakye K, Kiambi S, Koka E, Kabali E, Dorado-Garcia A, Amoah A, et al. The impacts of animal Health service providers on antimicrobial use attitudes and practices: an examination of poultry layer farmers in Ghana and Kenya. Antibiot (Basel). 2020;9(9):554. 10.3390/antibiotics9090554. 10.3390/antibiotics9090554 PMC7557566 32872381

[11] Borelli E, Ellis K, Tomlinson M, Hotchkiss E. Antimicrobial usage and resistance in Scottish dairy herds: a survey of farmers’ knowledge, behaviours and attitudes. BMC Vet Res. 2023;19(1):72. 10.1186/s12917-023-03625-0. 37208702 10.1186/s12917-023-03625-0 PMC10197045

[12] Caudell MA, Dorado-Garcia A, Eckford S, Creese C, Byarugaba DK, Afakye K, et al. Towards a bottom-up understanding of antimicrobial use and resistance on the farm: a knowledge, attitudes, and practices survey across livestock systems in five African countries. PLoS One. 2020;15(1):e0220274. 10.1371/journal.pone.0220274. 10.1371/journal.pone.0220274 PMC6980545 31978098

[13] Dandi SO, Abarike ED, Abobi SM, Doke DA, Lyche JL, Addo S, et al. Knowledge, attitudes, and practices of antibiotic use among small-, medium-, and large-scale Fish farmers of the stratum II of the Volta Lake of Ghana. Antibiot (Basel). 2024;13(7):582. 10.3390/antibiotics13070582. 10.3390/antibiotics13070582 PMC11273686 39061263

[14] Dhayal VS, Krishnan A, Rehman BU, Singh VP. Understanding knowledge and Attitude of farmers towards antibiotic use and antimicrobial resistance in Jhunjhunu district, Rajasthan India. Antibiot (Basel). 2023;12(12):1718. 10.3390/antibiotics12121718. 10.3390/antibiotics12121718 PMC10740745 38136752

[15] Diana A, Snijders S, Rieple A, Boyle LA. Why do Irish pig farmers use medications? Barriers for effective reduction of antimicrobials in Irish pig production. Ir Vet J. 2021;74(1):12. 10.1186/s13620-021-00193-3. 33941278 10.1186/s13620-021-00193-3 PMC8091703

[16] Doidge C, Ferguson E, Lovatt F, Kaler J. Understanding farmers’ naturalistic decision making around prophylactic antibiotic use in lambs using a grounded theory and natural language processing approach. Preventative Vet Med. 2021;186:105226. 10.1016/j.prevetmed.2020.105226. 10.1016/j.prevetmed.2020.105226 33276298

[17] Friedman DB, Kanwat CP, Headrick ML, Patterson NJ, Neely JC, Smith LU. Importance of prudent antibiotic use on dairy farms in South Carolina: a pilot project on farmers’ knowledge, attitudes and practices. Zoonoses Public Hlth. 2007;54(9–10):366–75. 10.1111/j.1863-2378.2007.01077.x. 10.1111/j.1863-2378.2007.01077.x 18035975

[18] Lekagul A, Tangcharoensathien V, Mills A, Rushton J, Yeung S. How antibiotics are used in pig farming: a mixed-methods study of pig farmers, feed mills and veterinarians in Thailand. BMJ Glob Health. 2020;5(2):e001918. 10.1136/bmjgh-2019-001918. 10.1136/bmjgh-2019-001918 PMC7050320 32180998

[19] Luu QH, Nguyen TLA, Pham TN, Vo NG, Padungtod P. Antimicrobial use in household, semi-industrialized, and industrialized pig and poultry farms in Viet Nam. Preventative Vet Med. 2021;189:105292. 10.1016/j.prevetmed.2021.105292. 10.1016/j.prevetmed.2021.105292 33621709

[20] Moore DA, Blackburn CC, Afema JA, Kinder DR, Sischo WM. Describing motivation for health and treatment decisions and communication choices of calf-care workers on western United States dairies. J Dairy Sci. 2021;104(3):3197–209. 10.3168/jds.2020-18669. 33455797 10.3168/jds.2020-18669

[21] Nuvey FS, Mensah GI, Zinsstag J, Hattendorf J, Fink G, Bonfoh B, et al. Management of diseases in a ruminant livestock production system: a participatory appraisal of the performance of veterinary services delivery, and utilization in Ghana. BMC Vet Res. 2023;19(1):237. 10.1186/s12917-023-03793-z. 37968624 10.1186/s12917-023-03793-z PMC10647120

[22] Ojo OE, Fabusoro E, Majasan AA, Dipeolu MA. Antimicrobials in animal production: usage and practices among livestock farmers in Oyo and Kaduna States of Nigeria. Trop Anim Health Prod. 2016;48(1):189–97. 10.1007/s11250-015-0939-8. 26526955 10.1007/s11250-015-0939-8

[23] Ozdikmenli Tepeli S. A survey of knowledge, attitude, and practices surrounding antimicrobial use by family dairy farmers to mastitis control. Preventative Vet Med. 2023;214:105904. 10.1016/j.prevetmed.2023.105904. 10.1016/j.prevetmed.2023.105904 36958150

[24] Patnaik NM, Gupta J, Meena BS. Field level study to understand dimensions of antimicrobial use in dairy farms of Punjab. IJDS. 2020;73(5):457–63. 10.33785/IJDS.2020.v73i05.011.

[25] Tasmim ST, Hasan MM, Talukder S, Mandal AK, Parvin MS, Ali MY, et al. Sociodemographic determinants of use and misuse of antibiotics in commercial poultry farms in Bangladesh. IJID Regions. 2023;7:146–58. 10.1016/j.ijregi.2023.01.001. 37082426 10.1016/j.ijregi.2023.01.001 PMC10112016

[26] Tran Thi Kim Chi TTKC, Clausen JH, Phan Thi Van PTV, Tersbol B, Dalsgaard A. Use practices of antimicrobials and other compounds by shrimp and fish farmers in Northern Vietnam. Aquacult Rep. 2017;7:40–47. 10.1016/j.aqrep.2017.05.003.

[27] Truong DB, Doan HP, Doan Tran VK, Nguyen VC, Bach TK, Rueanghiran C, et al. Assessment of drivers of antimicrobial usage in poultry farms in the Mekong Delta of Vietnam: a combined participatory Epidemiology and Q-Sorting approach. Front Vet Sci. 2019;6:84. 10.3389/fvets.2019.00084. 30968033 10.3389/fvets.2019.00084 PMC6442645

[28] Turkson PK. Use of drugs and antibiotics in poultry production in Ghana. Ghana J Agric Sci. 2009;41(1):unpaginated. 10.4314/gjas.v41i1.46142.

[29] Adam CJM, Fortané N, Ducrot C, Paul MC. Transition pathways toward the prudent use of antimicrobials: the case of free-range Broiler farmers in France. Front Vet Sci. 2020;7:548483. 10.3389/fvets.2020.548483. 33134347 10.3389/fvets.2020.548483 PMC7577212

[30] Bradford H, McKernan C, Elliott C, Dean M. Factors influencing pig farmers’ perceptions and attitudes towards antimicrobial use and resistance. Preventative Vet Med. 2022;208:105769. 10.1016/j.prevetmed.2022.105769. 10.1016/j.prevetmed.2022.105769 36240619

[31] Cobo-Angel C, LeBlanc SJ, Roche SM, Ritter C. A focus group study of Canadian Dairy farmers’ attitudes and Social Referents on antimicrobial use and antimicrobial resistance. Front Vet Sci. 2021;8:645221. 10.3389/fvets.2021.645221. 34212017 10.3389/fvets.2021.645221 PMC8239135

[32] Dankar I, Hassan H, Serhan M. Knowledge, attitudes, and perceptions of dairy farmers regarding antibiotic use: lessons from a developing country. J Dairy Sci. 2022;105(2):1519–32. 10.3168/jds.2021-20951. 34998539 10.3168/jds.2021-20951

[33] de Jong E, van der Velden I, Smid A-MC, Ida JA, Reyher KK, Kelton DF, et al. Dairy farmers’ considerations for antimicrobial treatment of clinical mastitis in British Columbia and Alberta, Canada. Front Vet Sci. 2024;11:1417958. 10.3389/fvets.2024.1417958. 39176396 10.3389/fvets.2024.1417958 PMC11340526

[34] Fischer K, Sjöström K, Stiernström A, Emanuelson U. Dairy farmers’ perspectives on antibiotic use: a qualitative study. J Dairy Sci. 2019;102(3):2724–37. 10.3168/jds.2018-15015. 30612802 10.3168/jds.2018-15015

[35] Hibbard R, Chapot L, Yusuf H, Ariyanto KB, Maulana KY, Febriyani W, et al. It’s a habit. They’ve been doing it for decades and they feel good and safe.”: a qualitative study of barriers and opportunities to changing antimicrobial use in the Indonesian poultry sector. PLoS One. 2023;18(9):e0291556. 10.1371/journal.pone.0291556. 10.1371/journal.pone.0291556 PMC10519599 37747889

[36] Huey S, Kavanagh M, Regan A, Dean M, McKernan C, McCoy F, et al. Engaging with selective dry cow therapy: understanding the barriers and facilitators perceived by Irish farmers. Ir Vet J. 2021;74(1):28. 10.1186/s13620-021-00207-0. 34686221 10.1186/s13620-021-00207-0 PMC8540178

[37] Ida JA, Wilson WM, Nydam DV, Gerlach SC, Kastelic JP, Russell ER, et al. Contextualized understandings of dairy farmers’ perspectives on antimicrobial use and regulation in Alberta, Canada. J Dairy Sci. 2023;106(1):547–64. 10.3168/jds.2021-21521. 36424321 10.3168/jds.2021-21521 PMC10957287

[38] Jannah N, Fahrunnisa F, Paramitadevi YV, Vibowo H, Kurniawan FA, NA, et al. Antibiotic utilization and its implications among ruminant farmers and stakeholders in sumbawa regency, Indonesia. Vet Med Int. 2024;2024(1):2024:6519659. 10.1155/vmi/6519659. 10.1155/vmi/6519659 PMC11661870 39712530

[39] Khan X, Lim RHM, Rymer C, Ray P. Fijian farmers’ Attitude and knowledge towards antimicrobial use and antimicrobial resistance in livestock production systems–A qualitative study. Front Vet Sci. 2022;9:838457. 10.3389/fvets.2022.838457. 35433900 10.3389/fvets.2022.838457 PMC9007610

[40] Landfried L, Barnidge E, Pithua P, Lewis R, Jacoby J, King C, et al. Antibiotic use on Goat farms: an Investigation of knowledge, attitudes, and behaviors of Missouri Goat farmers. Anim (Basel). 2018;8(11):198. 10.3390/ani8110198. 10.3390/ani8110198 PMC6262384 30404160

[41] Lekagul A, Tangcharoensathien V, Liverani M, Mills A, Rushton J, Yeung S. Understanding antibiotic use for pig farming in Thailand: a qualitative study. Antimicrob Resist Infect Control. 2021;10(1):3. 10.1186/s13756-020-00865-9. 33407887 10.1186/s13756-020-00865-9 PMC7789695

[42] Lim JM, Duong MC, Hsu LY, Tam CC. Determinants influencing antibiotic use in Singapore’s small-scale aquaculture sectors: a qualitative study. PLoS One. 2020;15(2):e0228701. 10.1371/journal.pone.0228701. 10.1371/journal.pone.0228701 PMC7041790 32097422

[43] Masud AA, Rousham EK, Islam MA, Alam M-U, Rahman M, Mamun AA, et al. Drivers of antibiotic use in poultry production in Bangladesh: dependencies and dynamics of a patron-client relationship. Front Vet Sci. 2020, February, 6(;7. 10.3389/fvets.2020.00078. 10.3389/fvets.2020.00078 PMC7058630 32185184

[44] Pate LA, Milne CE, McMorran R, Roberts DJ, Macrae AI. Factors influencing Scottish dairy farmers’ antibiotic use. Vet Rec. 2023;192(12):e2997. 10.1002/vetr.2997. 10.1002/vetr.2997 37183187

[45] Skjølstrup NK, Lastein DB, Jensen CS, Vaarst M. The antimicrobial landscape as outlined by Danish dairy farmers. J Dairy Sci. 2021;104(10):11147–64. 10.3168/jds.2021-20552. 34364645 10.3168/jds.2021-20552

[46] Swinkels JM, Hilkens A, Zoche-Golob V, Krömker V, Buddiger M, Jansen J, et al. Social influences on the duration of antibiotic treatment of clinical mastitis in dairy cows. J Dairy Sci. 2015;98(4):2369–80. 10.3168/jds.2014-8488. 25682148 10.3168/jds.2014-8488

[47] Thongyuan S, Tansakul N. Antimicrobial use on pig farms in Thailand: Farmer perceptions of use and resistance. Preventative Vet Med. 2024;230:106287. 10.1016/j.prevetmed.2024.106287. 10.1016/j.prevetmed.2024.106287 39059075

[48] Adam CJM, Fortané N, Coviglio A, Delesalle L, Ducrot C, Paul MC. Epidemiological assessment of the factors associated with antimicrobial use in French free-range broilers. BMC Vet Res. 2019;15(1):219. 10.1186/s12917-019-1970-1. 31253174 10.1186/s12917-019-1970-1 PMC6599332

[49] Aniume T, Khanal A, Browning JR, Leite-Browning ML, Kilonzo-Nthenge A. Influences of management practices, information sources, and awareness on use of antibiotics among small-scale goat and sheep farmers. Appl Anim Sci. 2023;39(5):317–29. 10.15232/aas.2023-02391.

[50] Backhans A, Sjölund M, Lindberg A, Emanuelson U. Antimicrobial use in Swedish farrow-to-finish pig herds is related to farmer characteristics. Porc Health Manag. 2016;2(1):18. 10.1186/s40813-016-0035-0. 10.1186/s40813-016-0035-0 PMC5382483 28405444

[51] Chowdhury S, Fournié G, Blake D, Henning J, Conway P, Hoque MA, et al. Antibiotic usage practices and its drivers in commercial chicken production in Bangladesh. PLoS One. 2022;17(10):e0276158. 10.1371/journal.pone.0276158. 10.1371/journal.pone.0276158 PMC9576089 36251714

[52] Chowdhury S, Rheman S, Debnath N, Delamare-Deboutteville J, Akhtar Z, Ghosh S, et al. Antibiotics usage practices in aquaculture in Bangladesh and their associated factors. One Health. 2022;15:100445. 10.1016/j.onehlt.2022.100445. 36277097 10.1016/j.onehlt.2022.100445 PMC9582543

[53] Cobo-Angel C, Gohar B, LeBlanc SJ. Values and Risk perception shape Canadian Dairy farmers’ attitudes toward prudent use of antimicrobials. Antibiot (Basel). 2022;11(5):550. 10.3390/antibiotics11050550. 10.3390/antibiotics11050550 PMC9137716 35625194

[54] Coyne L, Patrick I, Arief R, Benigno C, Kalpravidh W, McGrane J, et al. The costs, benefits and human behaviours for antimicrobial use in small commercial Broiler Chicken systems in Indonesia. Antibiot (Basel). 2020;9(4):154. 10.3390/antibiotics9040154. 10.3390/antibiotics9040154 PMC7235826 32244693

[55] Doidge C, Lima E, Lovatt F, Hudson C, Kaler J. From the other perspective: behavioural factors associated with Uk sheep farmers’ attitudes towards antibiotic use and antibiotic resistance. PLoS One. 2021;16(5):e0251439. 10.1371/journal.pone.0251439. 10.1371/journal.pone.0251439 PMC8159000 34043635

[56] Doyle E, Heller J, Norris JM. Factors influencing dairy cattle farmer use of antimicrobials on farms in New South Wales, Australia. Aust Vet J. 2022;100(12):587–95. 10.1111/avj.13209. 36173313 10.1111/avj.13209 PMC10086797

[57] Farhan M, Awan N, Kanwal A, Sharif F, Hayyat MU, Shahzad L, et al. Dairy farmers’ levels of awareness of antibiotic use in livestock farming in Pakistan. Humanit Soc Sci Commun. 2024;11(1):165. 10.1057/s41599-023-02518-9.

[58] Geta K, Kibret M. Knowledge, attitudes and practices of animal farm owners/workers on antibiotic use and resistance in amhara region, north western Ethiopia. Sci Rep. 2021;11(1):21211. 10.1038/s41598-021-00617-8. 34707158 10.1038/s41598-021-00617-8 PMC8551280

[59] Hassan MM, Kalam MA, Alim MA, Shano S, Nayem MRK, Badsha MR, et al. Knowledge, Attitude, and practices on antimicrobial use and antimicrobial resistance among commercial poultry farmers in Bangladesh. Antibiot (Basel). 2021;10(7):784. 10.3390/antibiotics10070784. 10.3390/antibiotics10070784 PMC8300693 34203195

[60] Hirwa EM, Mujawamariya G, Shimelash N, Shyaka A. Evaluation of cattle farmers’ knowledge, attitudes, and practices regarding antimicrobial use and antimicrobial resistance in Rwanda. PLoS One. 2024;19(4):e0300742. 10.1371/journal.pone.0300742. 10.1371/journal.pone.0300742 PMC11008905 38603685

[61] Isomura R, Matsuda M, Sugiura K. Analyzing pig farmers’ and veterinarians’ perceptions and intentions to reduce antimicrobial usage in Japan. J Retailing Vet Epidemiol. 2017;21(2):115–22. 10.2743/jve.21.115.

[62] Jones PJ, Marier EA, Tranter RB, Wu G, Watson E, Teale CJ. Factors affecting dairy farmers’ attitudes towards antimicrobial medicine usage in cattle in England and Wales. Preventative Vet Med. 2015;121(1–2):30–40. 10.1016/j.prevetmed.2015.05.010. 10.1016/j.prevetmed.2015.05.010 26123631

[63] Kemp SA, Pinchbeck GL, Fevre EM, Williams NJ. A cross-sectional survey of the knowledge, attitudes, and practices of antimicrobial users and providers in an area of high-density livestock-human population in Western Kenya. Front Vet Sci. 2021, September, 7(;8. 10.3389/fvets.2021.727365. 10.3389/fvets.2021.727365 PMC8490823 34621809

[64] Kigozi MM, Higenyi J. Evaluation of farmer’s knowledge and application of guidelines on use of veterinary antibiotics in layer poultry production in Mukono district, central Uganda. Livest Res Rural Devel. 2017;29(9): Article 176.

[65] Kisoo L, Muloi DM, Oguta W, Ronoh D, Kirwa L, Akoko J, et al. Practices and drivers for antibiotic use in cattle production systems in Kenya. One Health. 2023;17:100646. 10.1016/j.onehlt.2023.100646. 38024269 10.1016/j.onehlt.2023.100646 PMC10665206

[66] Nohrborg S, Nguyen-Thi T, Xuan HN, Lindahl J, Boqvist S, Järhult JD, et al. Understanding Vietnamese chicken farmers’ knowledge and practices related to antimicrobial resistance using an item response theory approach. Front Vet Sci. 2024;11:1319933. 10.3389/fvets.2024.1319933. 38645642 10.3389/fvets.2024.1319933 PMC11027563

[67] Nohrborg S, Dione MM, Winfred AC, Okello L, Wieland B, Magnusson U. Geographic and socioeconomic influence on knowledge and practices related to antimicrobial resistance among smallholder pig farmers in Uganda. Antibiot (Basel). 2022;11(2):251. 10.3390/antibiotics11020251. 10.3390/antibiotics11020251 PMC8868422 35203853

[68] Okello DM, Aliro T, Odongo W, Ndyomugyenyi EK, Owiny DO. Alone or a combination: ascertaining factors associated with choice of pig health management strategies amongst smallholder farmers in northern Uganda. Preventative Vet Med. 2022;199:105562. 10.1016/j.prevetmed.2021.105562. 10.1016/j.prevetmed.2021.105562 34953300

[69] Omolo JO, Omani R, Caudell MA, Kimani T, Kiambi S, Fasina FO, et al. Knowledge, attitudes, practices on antimicrobial use in animals among livestock sector stakeholders in Kenya. Vet Med Int. 2024;2024(1):2024: 8871774. 10.1155/2024/8871774. 10.1155/2024/8871774 PMC11599476 39606423

[70] Oyebanji BO. Use of antibiotics and knowledge of antibiotics resistance by selected farmers in Oyo town, Nigeria. UJAS. 2018;18(1):43–56. 10.4314/ujas.v18i1.4.

[71] Pham-Duc P, Cook MA, Cong-Hong H, Nguyen-Thuy H, Padungtod P, Nguyen-Thi H, et al. Knowledge, attitudes and practices of livestock and aquaculture producers regarding antimicrobial use and resistance in Vietnam. PLoS One. 2019;14(9):e0223115. 10.1371/journal.pone.0223115. 10.1371/journal.pone.0223115 PMC6760827 31553776

[72] Rahman MS, Ape T, Islam M, Chowdhury S. Perception of dairy farmers regarding antibiotic use and antimicrobial resistance in Bangladesh. SJA. 2021;37(4):1238–43. 10.17582/journal.sja/2021/37.4.1238.1243.

[73] Ratanapob N, Saengtienchai A, Rukkwamsuk RT. Knowledge, Attitude, and Practice of Thai Dairy farmers on the use of antibiotics. Vet Med Int. 2024;2024(1):5553760. 10.1155/2024/5553760. 38974506 10.1155/2024/5553760 PMC11226334

[74] Sadiq MB, Syed-Hussain SS, Ramanoon SZ, Saharee AA, Ahmad NI, Mohd Zin N, et al. Knowledge, attitude and perception regarding antimicrobial resistance and usage among ruminant farmers in Selangor, Malaysia. Preventative Vet Med. 2018;156:76–83. 10.1016/j.prevetmed.2018.04.013. 10.1016/j.prevetmed.2018.04.013 29891148

[75] Sawadogo A, Kagambèga A, Moodley A, Ouedraogo AA, Barro N, Dione DM. Knowledge, attitudes, and practices related to antibiotic use and antibiotic resistance among poultry farmers in Urban and Peri-Urban areas of Ouagadougou, Burkina Faso. Antibiot (Basel). 2023;12(1):133. 10.3390/antibiotics12010133. 10.3390/antibiotics12010133 PMC9854744 36671334

[76] Schneider S, Salm F, Vincze S, Moeser A, Petruschke I, Schmücker K, et al. Perceptions and attitudes regarding antibiotic resistance in Germany: a cross-sectoral survey amongst physicians, veterinarians, farmers and the general public. The J Antimicrob Chemother. 2018;73(7):1984–88. 10.1093/jac/dky100. 29590400 10.1093/jac/dky100

[77] Si R, Yao Y, Liu M. Effectiveness of information acquisition via the internet in standardizing the use of antimicrobials by hog farmers: insights from China. Agriculture. 2023;13(8):1586. 10.3390/agriculture13081586.

[78] Ström G, Boqvist S, Albihn A, Fernström LL, Andersson Djurfeldt A, Sokerya S, et al. Antimicrobials in small-scale urban pig farming in a lower middle-income country – arbitrary use and high resistance levels. Antimicrob Resist Infect Control. 2018;7(1):35. 10.1186/s13756-018-0328-y. 29541447 10.1186/s13756-018-0328-y PMC5842516

[79] van Asseldonk M, de Lauwere C, Bonestroo J, Bondt N, Bergevoet R, van Asseldonk M, et al. Antibiotics use versus profitability on sow farms in the Netherlands. Preventative Vet Med. 2020;178:104981. 10.1016/j.prevetmed.2020.104981. 10.1016/j.prevetmed.2020.104981 32279001

[80] Vasquez AK, Foditsch C, Dulièpre S-AC, Siler JD, Just DR, Warnick LD, et al. Understanding the effect of producers’ attitudes, perceived norms, and perceived behavioral control on intentions to use antimicrobials prudently on New York dairy farms. PLoS One. 2019;14(9):e0222442. 10.1371/journal.pone.0222442. 10.1371/journal.pone.0222442 PMC6738616 31509595

[81] Zwald AG, Ruegg PL, Kaneene JB, Warnick LD, Wells SJ, Fossler C, et al. Management practices and reported antimicrobial usage on conventional and organic dairy farms. J Dairy Sci. 2004;87(1):191–201. 10.3168/jds.S0022-0302(04)73158-6. 14765827 10.3168/jds.S0022-0302(04)73158-6

[82] Ekakoro JE, Caldwell M, Strand EB, Okafor CC. Drivers, alternatives, knowledge, and perceptions towards antimicrobial use among Tennessee beef cattle producers: a qualitative study. BMC Vet Res. 2019;15(1):16. 10.1186/s12917-018-1731-6. 30616648 10.1186/s12917-018-1731-6 PMC6323766

[83] Adebisi YA. Balancing the risks and benefits of antibiotic use in a globalized world: the ethics of antimicrobial resistance. Globalizat Health. 2023;19(1):27. 10.1186/s12992-023-00930-z. 10.1186/s12992-023-00930-z PMC10116465 37081463

[84] Bettinger B, Benneyan JC, Mahootchi T. Antibiotic stewardship from a decision-making, behavioral economics, and incentive design perspective. Appl Ergon. 2021;90:103242. 10.1016/j.apergo.2020.103242. 32861088 10.1016/j.apergo.2020.103242

[85] Edet UI. Agricultural communication for Addressing Climate change Challenges: understanding farmers’ Responses to Misinformation. RuralReview. 2024;8(1). 10.21083/ruralreview.v8i1.7938.

[86] Arbuckle JG Jr, Morton LW, Hobbs J. Understanding farmer perspectives on Climate change adaptation and mitigation: the roles of trust in sources of Climate information, Climate change beliefs, and perceived Risk. Environ Behav. 2015;47(2):205–34. 10.1177/0013916513503832. 25983336 10.1177/0013916513503832 PMC4359208

[87] Rust NA, Stankovics P, Jarvis RM, Morris-Trainor Z, de Vries JR, Ingram J, et al. Have farmers had enough of experts? Environ Manag. 2022;69(1):31–44. 10.1007/s00267-021-01546-y. 10.1007/s00267-021-01546-y PMC8503873 34633488

[88] Svensson C, Lind N, Reyher KK, Bard AM, Emanuelson U. Trust, feasibility, and priorities influence Swedish dairy farmers’ adherence and nonadherence to veterinary advice. J Dairy Sci. 2019;102(11):10360–68. 10.3168/jds.2019-16470. 31495620 10.3168/jds.2019-16470

[89] Pasteur K, Diana A, Yatcilla JK, Barnard S, Croney CC. Access to veterinary care: evaluating working definitions, barriers, and implications for animal welfare. Front Vet Sci. 2024;11:1335410. 10.3389/fvets.2024.1335410. 38304544 10.3389/fvets.2024.1335410 PMC10830634

[90] Groves CNH, Coe JB, Sutherland KA, Bauman C, Grant LE. Clients prefer collaborative decision-making with veterinarians regardless of appointment type. Javma. 2025;263(1):1–11. 10.2460/javma.24.06.0421. 39326456 10.2460/javma.24.06.0421

[91] Speksnijder DC, Graveland H, Eijck I, Schepers RWM, Heederik DJJ, Verheij TJM, et al. Effect of structural animal health planning on antimicrobial use and animal health variables in conventional dairy farming in the Netherlands. J Dairy Sci. 2017;100(6):4903–13. 10.3168/jds.2016-11924. 28390724 10.3168/jds.2016-11924

[92] Lekagul A, Kirivan S, Kaewkhankhaeng W, Khotchalai S, Mader R, Tangcharoensathien V. Voluntary optimisation of antimicrobial consumption in swine and poultry production in Thailand: a policy analysis. Front Vet Sci. 2024;11:1375127. 10.3389/fvets.2024.1375127. 39051011 10.3389/fvets.2024.1375127 PMC11267447

[93] Chen Y, Mandler T, Meyer-Waarden L. Three decades of research on loyalty programs: a literature review and future research agenda. J Educ Chang Bus Res. 2021;124:179–97. 10.1016/j.jbusres.2020.11.057.

[94] Rosário A, Casaca JA. Relationship marketing and customer retention - a systematic literature review. Stud Bus Econ. 2023;18(3):44–66. 10.2478/sbe-2023-0044.

[95] Hassan AM, Abdulkarim A, Seida A. Veterinary telemedicine: a new era for animal welfare. Open Vet J. 2024;14(4):952–61. 10.5455/OVJ.2024.v14.i4.2. 38808291 10.5455/OVJ.2024.v14.i4.2 PMC11128645

[96] Haverkate M, Evans M, Walter EJS. Antimicrobial prescribing during telemedicine appointments. Open Vet J. 2024;14(12):3625–29. 10.5455/OVJ.2024.v14.i12.43. 39927359 10.5455/OVJ.2024.v14.i12.43 PMC11799632

[97] Huang Z, Wan X, Zhou S, Yu M. Telemedicine in action: improving perceived healthcare accessibility in rural China. Health Care (Don Mills) Sci. 2025;4(3):215–24. 10.1002/hcs2.70017. 10.1002/hcs2.70017 PMC12185372 40568632

[98] National advanced skills and training program for rural Practice. https://srpc.ca/skills-and-training-program/ Accessed. 10.4103/cjrm.cjrm_4_23 37005986

[99] Government of Canada increases loan forgiveness for doctors and nurses working in under-served rural and remote communities. https://www.canada.ca/en/employment-social-development/news/2024/02/government-of-canada-increases-loan-forgiveness-for-doctors-and-nurses-working-in-under-served-rural-and-remote-communities.html Accessed.

[100] Bugeza J, Kankya C, Muleme J, Akandinda A, Sserugga J, Nantima N, et al. Participatory evaluation of delivery of animal health care services by community animal health workers in Karamoja region of Uganda. PLoS One. 2017;12(6):e0179110. 10.1371/journal.pone.0179110. 10.1371/journal.pone.0179110 PMC5464622 28594945

[101] Karamoja ADVSIN. UGANDA: a REVIEW. 2016. https://karamojaresilience.org/wp-content/uploads/2021/05/tufts_1704_veterinary_uganda_review_online.pdf). Accessed.

[102] Luseba D, Rwambo P. Review ofthe policy, regulatory and administrative framework for delivery oflivestock health products and services in Eastern and Southern Africa. In.; 2015.

[103] Goutard FL, Bordier M, Calba C, Erlacher-Vindel, Góchez, de Balogh K, et al. Antimicrobial policy interventions in food animal production in South East Asia. BMJ. 2017;j3544. 10.1136/bmj.j3544. 10.1136/bmj.j3544 PMC5598294 28874351

[104] Valgarðsson V, Jennings W, Stoker G, Bunting H, Devine D, McKay L, et al. A crisis of Political trust? Global trends in institutional trust from 1958 to 2019. Brit J Polit Sci. 2025, 55;55. 10.1017/s0007123424000498.

[105] Davis K, Nkonya E, Kato E, Mekonnen DA, Odendo M, Miiro R, et al. Impact of farmer Field schools on Agricultural productivity and poverty in East Africa. World Devel. 2012;40(2):402–13. 10.1016/j.worlddev.2011.05.019.

[106] Pho AT, Bates N, Snow A, Zhang A, Logan R, Dastur Z, et al. Asking sexual orientation and gender identity on health surveys: findings from cognitive interviews in the United States across sexual orientations and genders. SSM - Qualitative Res Health. 2023, 4;4:100344. 10.1016/j.ssmqr.2023.100344. 10.1016/j.ssmqr.2023.100344 PMC12369996 40852178

