# Supplementary material for: Identifying and mapping key relationships and communication pathways influencing farmers’ antibiotic use in production animals: a scoping review
Source: One Health Outlook. 2026 May 26;8:29. doi: 10.1186/s42522-026-00215-6 (PMC13202927; doi:10.1186/s42522-026-00215-6)
Supplement: Supplementary file 2 — Supplementary material 2 : Table S1 and Figures S1 & S2 [file 42522_2026_215_MOESM2_ESM.pdf]

Supplementary Annex

Containing: Table S1 and Figure S1 and S2
Table S2 is provided as a separate file

Table S1. Search Strings

Search String #1 (PubMed)	("Anti-Bacterial Agents"[Mesh] OR "Anti Bacterial" OR "Antibacterial" OR "Anti Bacterial" OR "Anti-bacterial" OR "Bacteriocidal" OR "Bacteriocide" OR "Anti-Infective Agents"[Mesh] OR "Anti-Infective" OR "Antiinfective" OR "Microbicide" OR "Anti-Microbial" OR "Antimicrobial" OR antibacterial OR "anti-bacterial" OR antibiotic OR "anti-biotic" OR "anti biotic" OR antimicrobial OR "anti-microbial" OR "Anti microbial" OR "Animal Feed"[Mesh] OR "Animal Feed" OR "Fodder" OR "Medicated Feed" OR "Medicated Fodder" OR "Feed additive" OR "veterinary drug") AND (Advice OR Advis* OR Influenc* OR Instruct* OR Trust OR Direct* OR Communcat* OR Network OR Inform* OR Information OR Relation* OR "Health Knowledge, Attitudes, Practice"[Mesh] OR "Decision Making"[Mesh] OR Decision OR Decide OR Decid* OR "Information Seeking Behavior"[Mesh] OR "Information Seeking" OR "Information Seeking Behaviors" OR "Awareness"[Mesh] OR Awareness OR "Situational Awarenesses" OR "Attitude"[Mesh] OR Attitude OR Opinion OR Sentiment OR "Practice Management, Veterinary"[Mesh] OR "Veterinary Practice Management" OR Practice OR "Knowledge"[Mesh] OR Knowledge OR Epistemology OR "Behavior and Behavior Mechanisms"[Mesh] OR Behaviour OR Behavior OR "Interprofessional Relations"[Mesh] OR "Interprofessional Relation" OR "Medical Etiquette" OR "Drug Misuse"[Mesh] OR "Drug Misuse" OR "Non-Prescription" OR "Non Prescription" OR "Non-Prescription Medicine" OR "Over-the-Counter" OR "Over the Counter" OR "Psychology"[Mesh] OR Psychology OR "Psychological Factors" OR Psychosocial) AND ("Farmers"[Mesh] OR Farmer OR Farmworkers OR Farmworker OR Farm Workers OR Farm Worker OR Agricultural Workers OR Agricultural Worker OR Ranchers OR Rancher OR "Cattle Producer" OR "Livestock Producer" OR "Swine Producer" OR "Poultry Producer")
Search String #2 (CAB Abstracts, SSPC, PsycINFO)	("Anti-Bacterial Agents" OR "Anti Bacterial" OR "Antibacterial" OR "Anti Bacterial" OR "Anti-bacterial" OR "Bacteriocidal" OR "Bacteriocide" OR "Anti-Infective Agents" OR "Anti-Infective" OR "Antiinfective" OR "Microbicide" OR "Anti-Microbial" OR "Antimicrobial" OR antibacterial OR "anti-bacterial" OR antibiotic OR "anti-biotic" OR "anti biotic" OR antimicrobial OR "anti-microbial" OR "Anti microbial" OR "Animal Feed" OR "Fodder" OR "Medicated Feed" OR "Medicated Fodder" OR "Feed additive" OR "veterinary drug") AND (Advice OR Advis* OR Influenc* OR Instruct* OR Trust OR Direct* OR Communcat* OR Network OR Inform* OR Information OR Relation* OR "Health Knowledge, Attitudes, Practice" OR "Decision Making" OR Decision OR Decide OR Decid* OR "Information Seeking

	Behavior" OR “Information Seeking” OR “Information Seeking Behaviors” OR Awareness OR “Situational Awarenesses” OR Attitude OR Opinion OR Sentiment OR "Practice Management, Veterinary" OR “Veterinary Practice Management” OR Practice OR Knowledge OR Epistemology OR “Behavior and Behavior Mechanisms" OR Behaviour OR Behavior OR “Interprofessional Relation” OR “Medical Etiquette” OR “Drug Misuse” OR “Non Prescription” OR “Non-Prescription Medicine” OR “Over-the-Counter” OR “Over the Counter” OR Psychology OR “Psychological Factors” OR Psychosocial) AND (Farmer OR Farmworkers OR Farmworker OR Farm Workers OR Farm Worker OR Agricultural Workers OR Agricultural Worker OR Ranchers OR Rancher OR “Cattle Producer” OR “Livestock Producer” OR “Swine Producer” OR “Poultry Producer”)
Search String #3 (PubMed)	(hetacillin OR ibafloxacin OR iclaprim OR imipenem OR ionophore* OR isepamicin OR isoniazid OR josamycin OR kanamycin OR ketolilde* OR kitasamycin OR lasalocid OR latamoxef OR levofloxacin OR lincomycin OR lincosamide* OR linezolid OR lipopeptide* OR lomefloxacin OR loracarbef OR lymecycline OR macrolide* OR maduramycin OR marbofloxacin OR mecillinam OR meropenem OR metacycline OR metampicillin OR methicillin OR meticillin OR metronidazole OR mezlocillin OR midecamycin OR miloxacin OR miocamycin OR minocycline OR mirosamycin OR monensin OR monobactam* OR morinamide OR moxifloxacin OR mupirocin OR nafcillin OR nalidixic acid OR narasin OR neomycin OR netilmicin OR nifurtoinol OR nitrofur* OR nitroimidazole* OR norfloxacin OR novobiocin OR ofloxacin OR oleandomycin OR orbifloxacin OR oritavancin OR OR mosulfathiazole OR ornidazole OR orthosomycin* OR oxacillin OR oxazolidinone* OR oxolinic acid OR oxytetracycline OR panipenem OR para-aminosalicylic acid OR paromomycin OR pazufloxacin OR pefloxacin OR penamecillin OR penethatamate OR penicillin* OR penimepicycline OR phenethicillin OR pheneticillin OR phenicol* OR phenoxypenicillin* OR phenoxymethylpenicillin OR phthalylsulfathiazole OR pipemidic acid OR piperacillin OR pirlimycin OR piromidic acid OR pivampicillin OR pivmecillinam OR pleuromutilin* OR polymixin* OR polymyxin* OR polypeptide* OR pristinamycin OR propicillin OR protionamide OR prulifloxacin OR pseudomonic acid* OR pyrazinamide OR pyrimethamine OR quinolone* OR quinoxaline* OR quinupristin OR retapamulin OR ribostamycin OR rifa* OR riminofenazine* OR rokitamycin OR rolitetracycline OR rosoxacin OR roxithromycin OR rufloxacin OR salfadoxine OR salinomycin OR semduramicin OR sisomicin OR sitafloxacin OR sodium aminosalicylate OR sparfloxacin OR spectinomycin OR spiramycin OR streptoduocin OR streptogramin* OR streptomycin OR sulbenicillin OR sulfachlORpyridazine OR sulfadi* OR sulphadi* OR sulfafurazole OR sulphafurazole OR sulfaisodimidine OR sulfisoxazole OR sulfon*OR sulphon* OR sulfaguanidine OR sulfam* OR sulfanilamide OR sulfalene OR sulfam* OR sulpham* OR sulfanilamide OR sulfap* OR

	sulphap* OR sulfaquinoxaline OR sulfath* sulphath* OR sultamicillin OR talampicillin OR teicoplanin OR telavancin OR telithromycin OR temafloxacin OR temocillin OR terdecamysin OR terizidone OR tetracycline* OR tetroxoprim OR thiamphenicol OR tiamulin OR ticarcillin OR tigecycline OR tildipirosin OR tilmicosin OR tinidazole OR tiocarlide OR tobicillin OR tobramycin OR trimethoprim OR troleandomycin OR trovafloxacin OR tulathromycin OR tylosin OR tylvalosin OR valnemulin OR vancomycin OR virginiamycin) AND (Advice OR Advis* OR Influenc* OR Instruct* OR Trust OR Direct* OR Communcat* OR Network OR Inform* OR Information OR Relation* OR "Health Knowledge, Attitudes, Practice"[Mesh] OR "Decision Making"[Mesh] OR Decision OR Decide OR Decid* OR OR "Information Seeking Behavior"[Mesh] OR “Information Seeking” OR “Information Seeking Behaviors” OR "Awareness"[Mesh] OR Awareness OR “Situational Awarenesses” OR "Attitude"[Mesh] OR Attitude OR Opinion OR Sentiment OR "Practice Management, Veterinary"[Mesh] OR “Veterinary Practice Management” OR Practice OR "Knowledge"[Mesh] OR Knowledge OR Epistemology OR “Behavior and Behavior Mechanisms"[Mesh] OR Behaviour OR Behavior OR "Interprofessional Relations"[Mesh] OR “Interprofessional Relation” OR “Medical Etiquette” OR "Drug Misuse"[Mesh] OR “Drug Misuse” OR “Non-Prescription” OR “Non Prescription” OR “Non-Prescription Medicine” OR “Over-the-Counter” OR “Over the Counter” OR "Psychology"[Mesh] OR Psychology OR “Psychological Factors” OR Psychosocial) AND ("Farmers"[Mesh] OR Farmer OR Farmworkers OR Farmworker OR Farm Workers OR Farm Worker OR Agricultural Workers OR Agricultural Worker OR Ranchers OR Rancher OR “Cattle Producer” OR “Livestock Producer” OR “Swine Producer” OR “Poultry Producer”)
Search String #4 (PubMed)	(aldesulfone OR amdinopenicillin* OR amikacin OR aminocyclitol* OR aminoglycoside* OR aminopenicillin* OR amoxicillin* OR ampicillin OR amphenicol* OR ansamycin* OR antipseudomonal OR antistaphylococcal OR apramycin OR arbekacin OR aspoxicillin OR avilamycin OR avoparcin OR azalide OR azidocillin OR azithromycin OR azlocillin OR aztreonam OR bacampicillin OR bacitracin OR baquiloprim OR bekanamycin OR benzylpenicillin OR biapenem OR bicozamycin OR bicyclomycin* OR brodimoprim OR calcium aminosalicylate OR capreomycin OR carbadox OR carbapenem* OR carbenicillin OR carboxypenicillin* OR carindacillin OR carumonam OR Cefa* OR Cefu* OR Cefp* OR Cefo* OR Cefm* OR Cefb* OR Cefd* OR Ceft* OR Cefi* OR Cefc* OR Cefe* OR Cefl* OR Cefs* OR Cefr*<br>OR ceph* OR chloramphenicol OR chlortetracycline OR cinoxacin OR ciprofloxacin OR clarithromycin OR clindamycin OR clofazimine OR clometocillin OR clomocycline OR cloxacillin OR colistin OR cyclic ester* OR cyclic polypeptide* OR cycloserine OR dalbavancin OR dalfopristin OR danofloxacin OR dapsone OR daptomycin OR demeclocycline OR

	diaminopyrimidine* OR dibekacin OR dicloxacillin OR difloxacin OR dirithromycin OR dihydrostreptomycin OR doripenem OR doxycycline OR dihydrofolate reductase inhibitOR* OR enoxacin OR enramycin OR enrofloxacin OR epicillin OR ertapenem OR erythromycin OR ethambutol OR ethionamide OR faropenem OR fleroxacin OR flomoxef OR florphenicol OR flucloxacillin OR flumeqin* OR fluoroquinolone* OR flurithromycin OR fosfomycin OR framycetin OR furaltadone OR furazolidone OR fusidic acid OR gamithromycin OR garenoxacin OR gatifloxacin OR gemifloxacin OR gentamicin OR glycopeptide* OR glycylcycline* OR gramicidin OR grepafloxacin) AND (Advice OR Advis* OR Influenc* OR Instruct* OR Trust OR Direct* OR Communcat* OR Network OR Inform* OR Information OR Relation* OR "Health Knowledge, Attitudes, Practice"[Mesh] OR "Decision Making"[Mesh] OR Decision OR Decide OR Decid* OR "Information Seeking Behavior"[Mesh] OR "Information Seeking" OR "Information Seeking Behaviors" OR "Awareness"[Mesh] OR Awareness OR "Situational Awarenesses" OR "Attitude"[Mesh] OR Attitude OR Opinion OR Sentiment OR "Practice Management, Veterinary"[Mesh] OR "Veterinary Practice Management" OR Practice OR "Knowledge"[Mesh] OR Knowledge OR Epistemology OR "Behavior and Behavior Mechanisms"[Mesh] OR Behaviour OR Behavior OR "Interprofessional Relations"[Mesh] OR "Interprofessional Relation" OR "Medical Etiquette" OR "Drug Misuse"[Mesh] OR "Drug Misuse" OR "Non-Prescription" OR "Non Prescription" OR "Non-Prescription Medicine" OR "Over-the-Counter" OR "Over the Counter" OR "Psychology"[Mesh] OR Psychology OR "Psychological Factors" OR Psychosocial) AND ("Farmers"[Mesh] OR Farmer OR Farmworkers OR Farmworker OR Farm Workers OR Farm Worker OR Agricultural Workers OR Agricultural Worker OR Ranchers OR Rancher OR "Cattle Producer" OR "Livestock Producer" OR "Swine Producer" OR "Poultry Producer")
Search String #5 (CAB Abstracts, SSPC, PsycINFO)	(hetacillin OR ibafloxacin OR iclaprim OR imipenem OR ionophORe* OR isepamicin OR isoniazid OR josamycin OR kanamycin OR ketolilde* OR kitasamycin OR lasalocid OR latamoxef OR levofloxacin OR lincomycin OR lincosamide* OR linezolid OR lipopeptide* OR lomefloxacin OR loracarbef OR lymecycline OR macrolide* OR maduramycin OR marbofloxacin OR mecillinam OR meropenem OR metacycline OR metampicillin OR methicillin OR meticillin OR metronidazole OR mezlocillin OR midecamycin OR miloxacin OR miocamycin OR minocycline OR mirosamycin OR monensin OR monobactam* OR morinamide OR moxifloxacin OR mupirocin OR nafcillin OR nalidixic acid OR narasin OR neomycin OR netilmicin OR nifurtoinol OR nitrofur* OR nitroimidazole* OR norfloxacin OR novobiocin OR ofloxacin OR oleandomycin OR orbifloxacin OR oritavancin OR ORmosulfathiazole OR ORnidazole OR orthosomycin* OR oxacillin OR oxazolidinone* OR oxolinic acid OR oxytetracycline OR panipenem OR para-aminosalicylic acid OR paromomycin OR pazufloxacin OR pefloxacin OR

	penamecillin OR penethatamate OR penicillin* OR penimepicycline OR phenethicillin OR pheneticillin OR phenicol* OR phenoxypenicillin* OR phenoxymethylpenicillin OR phthalylsulfathiazole OR pipemidic acid OR piperacillin OR pirlimycin OR piromidic acid OR pivampicillin OR pivmecillinam OR pleuromutilin* OR polymixin* OR polymyxin* OR polypeptide* OR pristinamycin OR propicillin OR protionamide OR prulifloxacin OR pseudomonic acid* OR pyrazinamide OR pyrimethamine OR quinolone* OR quinoxaline* OR quinupristin OR retapamulin OR ribostamycin OR rifa* OR riminofenazine* OR rokitamycin OR rolitetracycline OR rosoxacin OR roxithromycin OR rufloxacin OR salfadoxine OR salinomycin OR semduramicin OR sisomicin OR sitafloxacin OR sodium aminosalicylate OR sparfloxacin OR spectinomycin OR spiramycin OR streptoduocin OR streptogramin* OR streptomycin OR sulbenicillin OR sulfachlORpyridazine OR sulfadi* OR sulphadi* OR sulfafurazole OR sulphafurazole OR sulfaisodimidine OR sulfisoxazole OR sulfon*OR sulphon* OR sulfaguanidine OR sulfam* OR sulfanilamide OR sulfalene OR sulfam* OR sulpham* OR sulfanilamide OR sulfap* OR sulphap* OR sulfaquinoxaline OR sulfath* sulphath* OR sultamicillin OR talampicillin OR teicoplanin OR telavancin OR telithromycin OR temafloxacin OR temocillin OR terdecamysin OR terizidone OR tetracycline* OR tetroxoprim OR thiamphenicol OR tiamulin OR ticarcillin OR tigecycline OR tildipirosin OR tilmicosin OR tinidazole OR tiocarlide OR tobicillin OR tobramycin OR trimethoprim OR troleandomycin OR trovafloxacin OR tulathromycin OR tylosin OR tylvalosin OR valnemulin OR vancomycin OR virginiamycin) AND (Advice OR Advis* OR Influenc* OR Instruct* OR Trust OR Direct* OR Communcat* OR Network OR Inform* OR Information OR Relation* OR "Health Knowledge, Attitudes, Practice" OR "Decision Making" OR Decision OR Decide OR Decid* OR "Information Seeking Behavior" OR “Information Seeking” OR “Information Seeking Behaviors” OR Awareness OR “Situational Awarenesses” OR Attitude OR Opinion OR Sentiment OR "Practice Management, Veterinary" OR “Veterinary Practice Management” OR Practice OR Knowledge OR Epistemology OR “Behavior and Behavior Mechanisms" OR Behaviour OR Behavior OR “Interprofessional Relation” OR “Medical Etiquette” OR “Drug Misuse” OR “Non Prescription” OR “Non-Prescription Medicine” OR “Over-the-Counter” OR “Over the Counter” OR Psychology OR “Psychological Factors” OR Psychosocial) AND (Farmer OR Farmworkers OR Farmworker OR Farm Workers OR Farm Worker OR Agricultural Workers OR Agricultural Worker OR Ranchers OR Rancher OR “Cattle Producer” OR “Livestock Producer” OR “Swine Producer” OR “Poultry Producer”)
Search String #6 (CAB Abstracts,	(aldesulfone OR amdinopenicillin* OR amikacin OR aminocyclitol* OR aminoglycoside* OR aminopenicillin* OR amoxicillin* OR ampicillin OR amphenicol* OR ansamycin* OR antipseudomonal OR antistaphylococcal OR apramycin OR arbekacin OR aspoxicillin OR avilamycin OR avoparcin OR

SSPC, PsycINFO)	azalide OR azidocillin OR azithromycin OR azlocillin OR aztreonam OR bacampicillin OR bacitracin OR baquiloprim OR bekanamycin OR benzylpenicillin OR biapenem OR bicozamycin OR bicyclomycin* OR brodimoprim OR calcium aminosalicylate OR capreomycin OR carbadox OR carbapenem* OR carbenicillin OR carboxypenicillin* OR carindacillin OR carumonam OR cef* OR ceph* OR chlORamphenicol OR chlORtetracycline OR cinoxacin OR ciprofloxacin OR clarithromycin OR clindamycin OR clofazimine OR clometocillin OR clomocycline OR cloxacillin OR colistin OR cyclic ester* OR cyclic polypeptide* OR cycloserine OR dalbavancin OR dalfopristin OR danofloxacin OR dapsone OR daptomycin OR demeclocycline OR diaminopyrimidine* OR dibekacin OR dicloxacillin OR difloxacin OR dirithromycin OR dihydrostreptomycin OR doripenem OR doxycycline OR dihydrofolate reductase inhibitOR* OR enoxacin OR enramycin OR enrofloxacin OR epicillin OR ertapenem OR erythromycin OR ethambutol OR ethionamide OR faropenem OR fleroxacin OR flomoxef OR florphenicol OR flucloxacillin OR flumeqin* OR fluoroquinolone* OR flurithromycin OR fosfomycin OR framycetin OR furaltadone OR furazolidone OR fusidic acid OR gamithromycin OR garenoxacin OR gatifloxacin OR gemifloxacin OR gentamicin OR glycopeptide* OR glycylcycline* OR gramicidin OR grepafloxacin) AND (Advice OR Advis* OR Influenc* OR Instruct* OR Trust OR Direct* OR Communcat* OR Network OR Inform* OR Information OR Relation* OR "Health Knowledge, Attitudes, Practice" OR "Decision Making" OR Decision OR Decide OR Decid* OR "Information Seeking Behavior" OR “Information Seeking” OR “Information Seeking Behaviors” OR Awareness OR “Situational Awarenesses” OR Attitude OR Opinion OR Sentiment OR "Practice Management, Veterinary" OR “Veterinary Practice Management” OR Practice OR Knowledge OR Epistemology OR “Behavior and Behavior Mechanisms" OR Behaviour OR Behavior OR “Interprofessional Relation” OR “Medical Etiquette” OR “Drug Misuse” OR “Non Prescription” OR “Non-Prescription Medicine” OR “Over-the-Counter” OR “Over the Counter” OR Psychology OR “Psychological Factors” OR Psychosocial) AND (Farmer OR Farmworkers OR Farmworker OR Farm Workers OR Farm Worker OR Agricultural Workers OR Agricultural Worker OR Ranchers OR Rancher OR “Cattle Producer” OR “Livestock Producer” OR “Swine Producer” OR “Poultry Producer”)

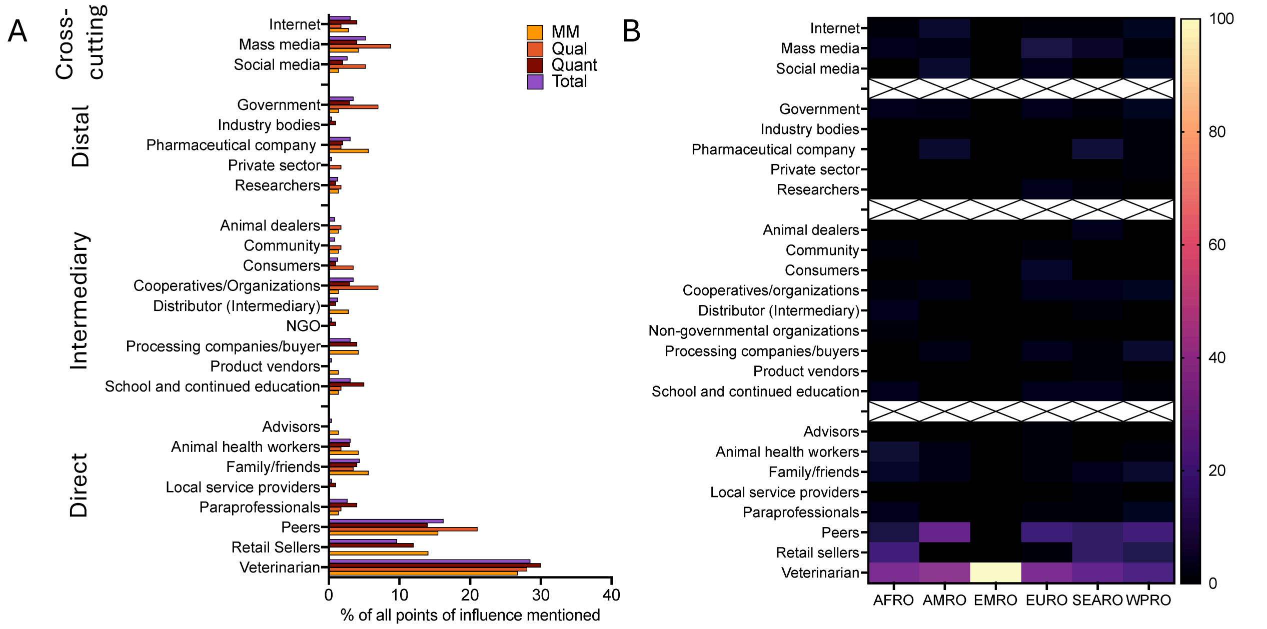

Fig. S1. Percentage mention of point of influence across all coded instances, by study type and across all studies included B. Heatmap showing the percentage mention of point of influence by number of total studies within a specific WHO region AFRO-African Region (N=51), AMRO – Region of the Americas (N=31), EMRO – Eastern Mediterranean Region (N=1), EURO- European Region (N=52), SEARO- South-East Asian Region (N=49), WPRO – Western Pacific Region (N=44). Points of influence are sorted by directness of interaction. MM = mixed methods (N=71), Qual =qualitative (N=57), Quant=quantitative (N=100), Total = all study types (N=228)

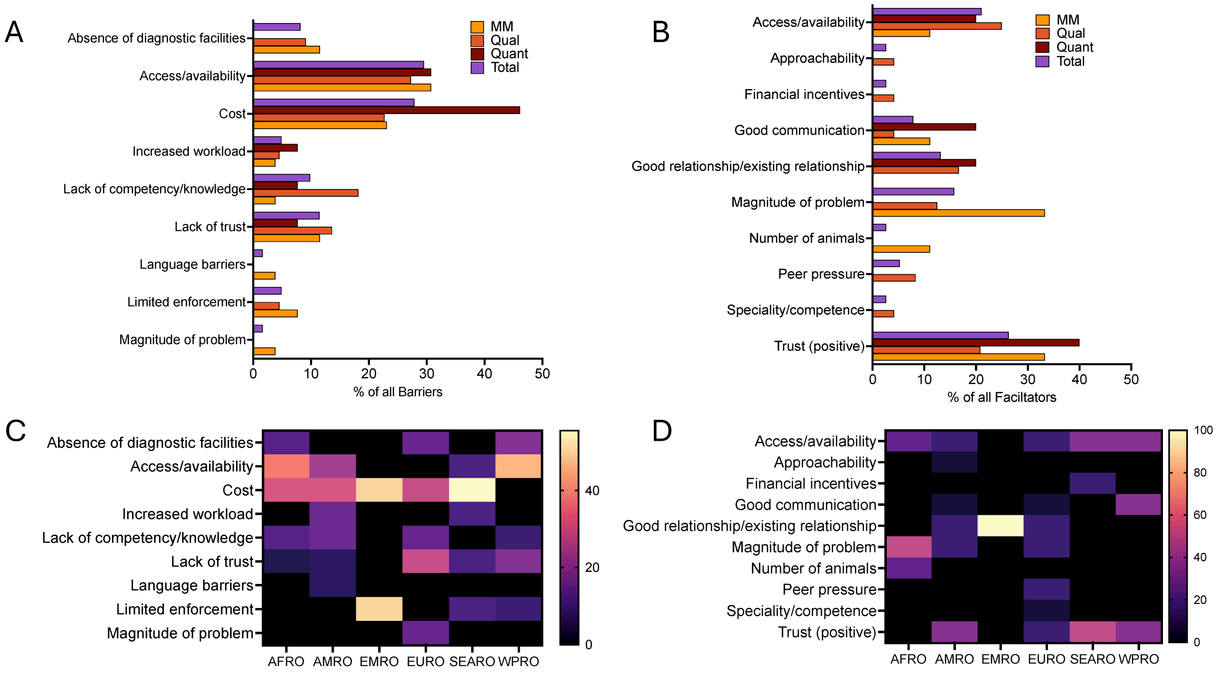

Fig. 5 A. Percentage mention of barrier described across all coded instances by study type (MM- N=26, Qual- N=,22, Quant- N=13) and as a percentage of total studies (N=61) B. Percentage mention of facilitator across all coded instances, by study type (MM- N=9, Qual- N=24, Quant- N=5) and as percentage of the total studies (N=38) C. Heatmap showing % barriers mentioned among all coded instances WHO region (AFRO- N=16, AMRO- N=13, EMRO-N=2, EURO-N=7, SEARO-N=9, WPRO-N=11) D. Heatmap showing percentage mentioned of facilitator among all coded instances by WHO region(AFRO- N=4, AMRO- N=12, EMRO-N=1, EURO-N=12, SEARO-N=6, WPRO-N=3). AFRO-African Region. AMRO – Region of the Americas, EMRO – Eastern Mediterranean Region, EURO- European Region, SEARO- South-East Asian Region, WPRO – Western Pacific Region. MM = mixed methods, Qual =qualitative, Quant=quantitative, Total = all study types
